# A Lipid‐Sensitive Spider Peptide Toxin Exhibits Selective Anti‐Leukemia Efficacy through Multimodal Mechanisms

**DOI:** 10.1002/advs.202404937

**Published:** 2024-07-04

**Authors:** Peng Zhang, Wu Luo, Zixin Zhang, Mingchong Lv, Longkang Sang, Yuhan Wen, Lingxiang Wang, Changhao Ding, Kun Wu, Fengjiao Li, Yueqi Nie, Jiaoyue Zhu, Xiaofeng Liu, Yan Yi, Xiaofeng Ding, Youlin Zeng, Zhonghua Liu

**Affiliations:** ^1^ The National & Local Joint Engineering Laboratory of Animal Peptide Drug Development College of Life Sciences Hunan Normal University Changsha Hunan 410081 China; ^2^ Peptide and Small Molecule Drug R&D Platform, Furong Laboratory Changsha Hunan 410081 China; ^3^ Institute of Interdisciplinary Studies Hunan Normal University Changsha 410081 China; ^4^ College of Biology Hunan University Changsha Hunan 410082 China; ^5^ Department of Hematology The Second Xiangya Hospital Central South University Changsha Hunan 410011 China; ^6^ Department of Hematology The Third Affiliated Hospital of Southern Medical University Southern Medical University Guangzhou 510630 China; ^7^ The National and Local Joint Engineering Laboratory for New Petrochemical Materials and Fine Utilization of Resources Hunan Normal University Changsha Hunan 410081 China

**Keywords:** anticancer peptide, apoptosis, cell cycle arrest, ferroptosis, leukemia, PI3K‐AKT‐mTOR signaling pathway

## Abstract

Anti‐cancer peptides (ACPs) represent a promising potential for cancer treatment, although their mechanisms need to be further elucidated to improve their application in cancer therapy. Lycosin‐I, a linear amphipathic peptide isolated from the venom of *Lycosa singorensis*, shows significant anticancer potential. Herein, it is found that Lycosin‐I, which can self‐assemble into a nanosphere structure, has a multimodal mechanism of action involving lipid binding for the selective and effective treatment of leukemia. Mechanistically, Lycosin‐I selectively binds to the K562 cell membrane, likely due to its preferential interaction with negatively charged phosphatidylserine, and rapidly triggers membrane lysis, particularly at high concentrations. In addition, Lycosin‐I induces apoptosis, cell cycle arrest in the G1 phase and ferroptosis in K562 cells by suppressing the PI3K‐AKT‐mTOR signaling pathway and activating cell autophagy at low concentrations. Furthermore, intraperitoneal injection of Lycosin‐I inhibits tumor growth of K562 cells in a nude mouse xenograft model without causing side effects. Collectively, the multimodal effect of Lycosin‐I can provide new insights into the mechanism of ACPs, and Lycosin‐I, which is characterized by high potency and specificity, can be a promising lead for the development of anti‐leukemia drugs.

## Graphical abstract

This study demonstrates a multimodal mechanism of spider toxin Lycosin‐I, which exhibits selective and effective treatment of leukemia. Lycosin‐I exhibits membrane lysis, particularly at high concentrations and triggers apoptosis, cell cycle arrest and ferroptosis by suppressing PI3K‐AKT‐mTOR signaling pathway. These findings deepen the understanding of the inhibitory mechanisms of anticancer peptides and Lycosin‐I can be a promising lead for leukemia therapy.

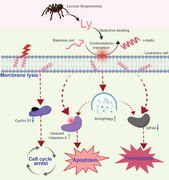

## 1 Introduction

Leukemia comprises a heterogeneous group of diseases characterized by the malignant clonal proliferation of blood progenitor cells. According to the American Cancer Society (ACS), an estimated 61,090 new cases of leukemia were reported in 2021.^[^ ^1^ ^]^ Significant progress has been made in the treatment of leukemia in recent years, including advances in combination chemotherapy, stem cell transplantation, and targeted therapies etc.^[^ ^2^ ^]^ Chemotherapy, as the most commonly used therapy, plays a crucial role in the advancement of leukemia treatment. Chemotherapeutic agents such as vincristine, daunorubicin, and prednisone are currently used as the predominant clinical regimens.^[^ ^3^, ^4^ ^]^ However, their efficacy is limited by drug resistance and high toxicity.^[^ ^5^, ^6^ ^]^ Therefore, there is an urgent need to explore more effective and less toxic treatments for leukemia.

Anti‐cancer peptides (ACPs) represent a novel therapeutic approach against tumor cells.^[^ ^7^ ^]^ Compared to antibodies and small molecules, ACPs have advantages such as high selectivity and the ability to rapidly induce cell death without developing drug resistance, making them a preferred choice for therapeutics.^[^ ^8^ ^]^ Animal venoms are complex mixtures of bioactive chemicals, especially disulfide‐rich proteins and peptides, with characteristics of high potency, specificity, and stability, making them promising therapeutic candidates.^[^ ^9^, ^10^ ^]^ In particular, animal venoms, rich in components, especially proteins and peptides, have significant anticancer potential, providing new opportunities for cancer treatment. Venom peptides (VPs), rich components in venom, exhibit high specificity and selectivity toward cancer cells, affecting cell proliferation, invasion, migration, and angiogenesis, as well as modulating immune responses.^[^ ^11^ ^]^ While membrane lysis, which has been shown to be regulated by the physicochemical parameters of the peptide,^[^ ^12^ ^]^ is considered the main mechanism of ACP‐induced cancer cell death, further research has revealed diverse mechanisms of action for ACPs.^[^ ^13^, ^14^ ^]^ For instance, *Naja naja* venom‐derived cardiotoxin III induces apoptosis in the human leukemia cell line K562 via a reactive oxygen species (ROS)‐independent pathway of mitochondrial dysfunction. This involves the upregulation of BAX and endonuclease G proteins, while the anti‐apoptotic Bcl‐XL protein is downregulated.^[^ ^15^ ^]^ In addition, peptide components in bee venom, such as melittin, induce apoptosis by downregulating ERK and AKT signaling pathways in U937 cells.^[^ ^16^ ^]^ Scorpion venom‐derived peptides such as Smp24 lead to cytoskeleton reorganization, accumulation of ROS, mitochondrial dysfunction, and alterations in the PI3K‐AKT‐mTOR and MAPK signaling pathways in HepG2 cells.^[^ ^17^ ^]^ However, the systemic mechanism of ACPs, especially novel mechanisms, remains to be elucidated. This holds significant importance to advance their application in cancer therapy.

Lycosin‐I is an anticancer peptide isolated from the venom of *Lycosa singorensis*. Our previous studies have demonstrated that Lycosin‐I can inhibit the growth of cancer and microbial cells.^[^ ^18^, ^19^, ^20^, ^21^ ^]^ Moreover, Lycosin‐I has been authorized a number of invention patents, showing good prospects for anticancer drug development, and further mechanistic studies on Lycosin‐I will greatly promote its clinical application. In this study, we further demonstrated that Lycosin‐I can form a nanosphere structure and exhibits lipid‐sensitive selective anti‐leukemia activity. This observation inspired us to further investigate the effect and mechanism of Lycosin‐I. For this purpose, fluorescence and cell morphology studies demonstrated the ability of Lycosin‐I to rapidly lyse the cell membrane of K562 cells, and clarified the cause of its selective effect on leukemia cells. Furthermore, transcriptomics and Western blot analysis indicated that Lycosin‐I can induce cell apoptosis and inhibit cell proliferation by suppressing the PI3K‐AKT‐mTOR signaling pathway. Interestingly, Lycosin‐I can also induce ferroptosis of K562 cells by disrupting iron homeostasis and inducing lipid peroxidation. Animal studies demonstrated that Lycosin‐I exhibits good efficacy and safety in vivo. In summary, Lycosin‐I works via four mechanisms of action: membrane lysis, cell cycle arrest, apoptosis, and ferroptosis, thus offering potential lead molecules and new ideas for leukemia therapy.

## 2 Results

### 2.1 Lycosin‐I Shows the Lipid‐Sensitive Conformational Transition

**Figure 1:**
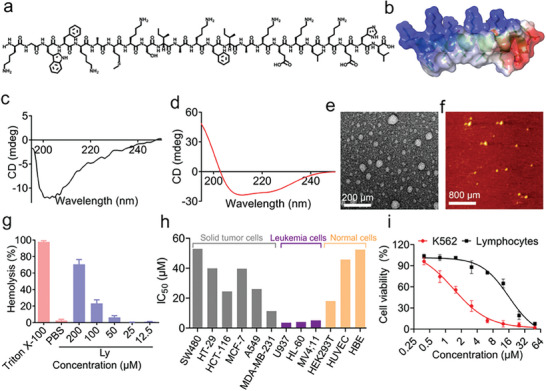
Lycosin‐I exhibits a potent and selective cytotoxic activity against leukemia cells through a lipid‐sensitive conformational transition. a) Chemical structures of the peptide Lycosin‐I. b) The computer simulation of the secondary structure model of Lycosin‐I predicted by the pymol‐open‐source. c) CD spectra of Lycosin‐I (50 µm) in PBS (7.4). d) CD spectra of Lycosin‐I (50 µm) in POPC/POPS (3:1). e,f) TEM (e) and AFM (f) images of the assemblies of 100 µM Lycosin‐I. g) Hemolytic activity of Lycosin‐I against human erythrocytes. h) The IC_50_ values of Lycosin‐I for the solid tumor cell lines MDA‐MB‐231, MCF‐7, A549, HCT116, HT‐29, and SW480, for human leukemia cell lines (MV4;11, HL‐60, and U937), and for non‐cancer cell lines (HUVEC, HBE, and HEK293T cells) were determined by CCK‐8 assay. i) The dose‐dependent response of Lycosin‐I to K562 cells and isolated normal human lymphocytes. Values are expressed as mean ± S.E.M.; *n* = 6 for each group.

Lycosin‐I, isolated from the venom of *Lycosa singorensis*, consists of 23 amino acid residues (**Figure** **1**a; Figures S1 and S2, Supporting Information). Similar to many α‐helical anticancer and antimicrobial peptides, Lycosin‐I has a linear amphipathic α‐helical conformation (Figure 1b; Figures S3 and S4, Supporting Information). Circular dichroism (CD) spectroscopy was used to analyze the secondary structure of Lycosin‐I in PBS (pH 7.4) and lipid vesicles formed by POPC/POPS, serving as model membranes (Figure S5, Supporting Information). In PBS, Lycosin‐I displayed a negative band at 200 nm (Figure 1c), indicating a random coil conformation. Intriguingly, Lycosin‐I exhibited the characteristic helical conformation in the presence of lipid vesicles, as evidenced by the double minima at 208 and 222 nm (Figure 1d). These results emphasize the conformational transition of Lycosin‐I from random coils to helices, which is facilitated by binding to the lipid bilayer. Morphological studies performed by transmission electron microscopy (TEM) revealed the formation of nanospheres with a diameter of 30–50 nm by Lycosin‐I (Figure 1e), an observation that was further confirmed by atomic force microscopy (AFM) (Figure 1f).

### 2.2 Lycosin‐I Effectively and Selectively Inhibits the Growth of Leukemia Cells

Lycosin‐I exhibits relatively low hemolytic activity, with a half‐hemolytic concentration of ≈180 µm (Figure 1g). Consistent with previous studies, Lycosin‐I demonstrated the ability to inhibit the viability of several human solid tumor cell lines, including MDA‐MB‐231, MCF‐7, A549, HCT116, HT‐29, and SW480, with IC_50_ values mostly between 20 and 50 µm (Figure 1h; Figure S6a, Supporting Information). We further investigated the inhibitory effect of Lycosin‐I on hematologic malignancies. Human leukemia cell lines, including K562, MV4;11, HL‐60, and U937, were treated with Lycosin‐I for 24 h. Lycosin‐I displayed a broad spectrum of stronger inhibitory activity in these leukemia cells compared to solid tumor cells, with IC_50_ values ranging from 1.66 to 4.2 µm (Figure 1h,i; Figure S6b, Supporting Information). Simultaneously, we observed that normal cells, including non‐cancer cell lines (HUVEC, HBE, and HEK293T cells) and isolated human lymphocytes, showed relatively low sensitivity to Lycosin‐I (Figure 1h,i; Figure S6b, Supporting Information). For instance, the IC_50_ value for K562 cells, the most sensitive cell line to Lycosin‐I in our assays, was determined to be 1.66 µm, whereas it was 15.8 µm for isolated human lymphocytes (Figure 1i), representing an almost eight‐fold selectivity for K562 cells.

To further evaluate the anti‐leukemia effect of Lycosin‐I, clinical leukemia cells were isolated from the bone marrow (BM) and peripheral blood (PB) of 21 diagnosed leukemia patients. The IC_50_ values of Lycosin‐I on leukemia cells isolated from the BM of 10 patients ranged from 2.41 to 7.01 µm, while the values on leukemia cells isolated from the PB of 11 patients ranged from 3.51 to 20.71 µm (**Table** **1**; and Figure S7, Supporting Information).

**Table 1:** The IC_50_ values of Lycosin‐I against clinically isolated leukemia cells.

Sample	Age/Sex	Disease	Source	IC_50_ [µm]
1	43/M	MDS/ALL	PB	2.86
2	41/M	MDS	BM	3.52
3	60/F	MDS	BM	4.87
4	35/M	CML	PB	1.77
5	40/F	CML	PB	3.81
6	60/M	AML	BM	3.20
7	56/M	AML	PB	17.1
8	76/M	AML	BM	4.58
9	55/M	AML	BM	4.92
10	57/F	ALL	BM	6.58
11	50/F	ALL	BM	6.00
12	40/M	CLL	PB	8.73
13	33/F	MPAL	PB	20.9
14	30/F	CML	PB	10.1
15	50/M	MPAL	PB	13.0
16	43/M	MDS/ALL	BM	2.42
17	58/M	CLL	BM	3.77
18	51/M	AML	PB	10.78
19	61/M	AML	BM	4.56
20	43/F	CML	PB	7.85
21	60/M	CML	PB	6.77

F: Female; M: Male. Clinical leukemia cells were isolated from BM and PB of 21 patients diagnosed with leukemia, including 6 patients with chronic myeloid leukemia (CML), 6 patients with acute myeloid leukemia (AML), 2 patients with chronic lymphocytic leukemia (CLL), 3 patients with acute lymphocytic leukemia (ALL), 2 patients with myelodysplastic syndrome (MDS) and 2 patients with mixed phenotype acute leukemia (MPAL). Freshly isolated cells were incubated with Lycosin‐I for 24 h and cell viability was determined using the CCK‐8 assay.

### 2.3 Phosphatidylserine Contributes to the Selective Binding and Anti‐Leukemia Activity of Lycosin‐I

**Figure 2:**
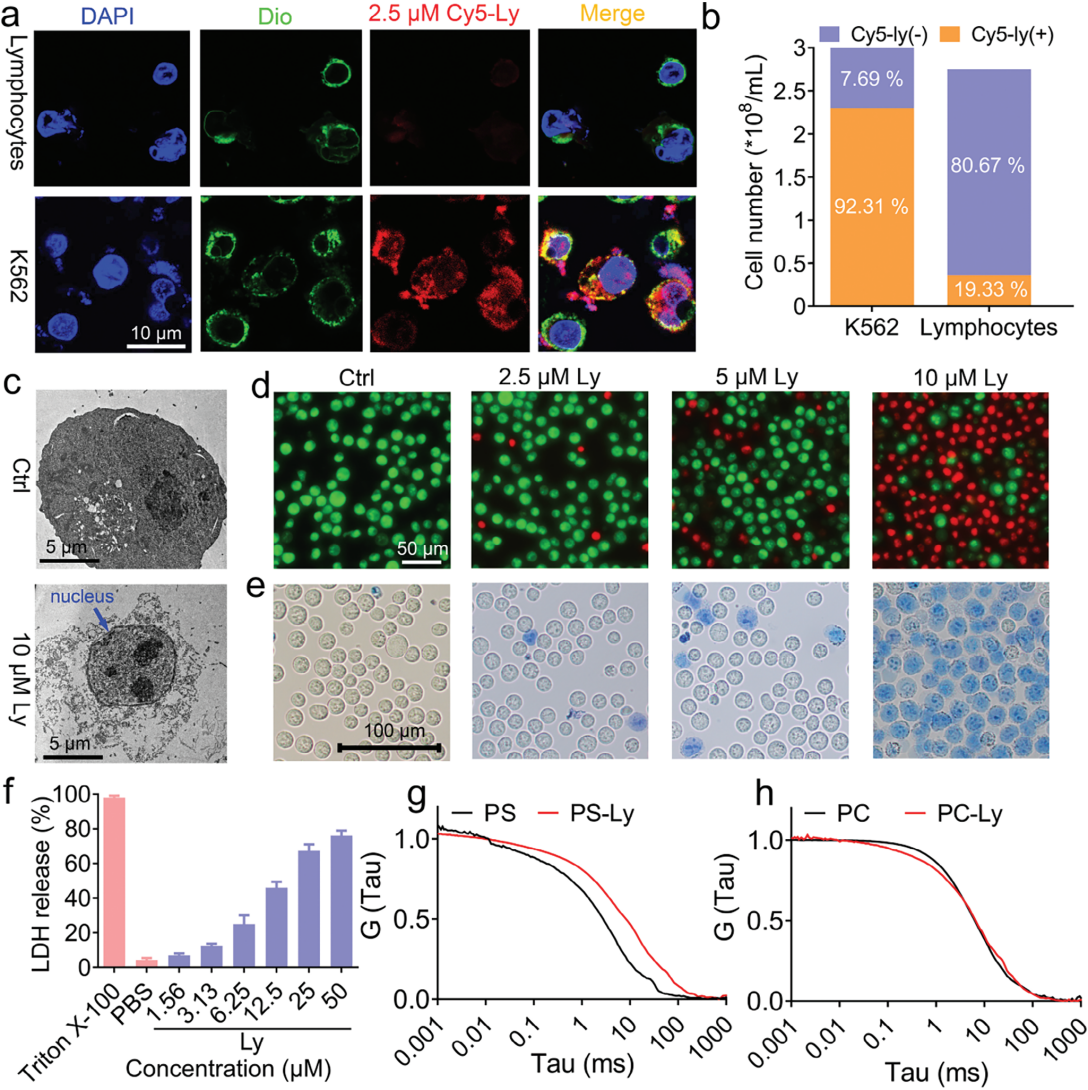
The ability of Lycosin‐I to bind cell membranes and its activity to rapidly destroy them. a) The distribution of Lycosin‐I in K562 cells or isolated lymphocytes was examined by confocal laser scanning microscopy. Cells were treated with Cy5‐ly at 2.5 µm for 1 h. b) Quantitative fluorescence analysis was performed based on the data from (a). c) TEM images of K562 cells after treatment with 10 µm Lycosin‐I for 1 h. d,e) SYTO9/PI double staining (d) and trypan blue staining (e) after K562 cells were treated with 2.5, 5, and 10 µm Lycosin‐I for 1 h, respectively. f) Measurement of LDH leakage after K562 cells were treated with Lycosin‐I at different concentrations for 1 h. PBS and Triton X‐100 represent the negative and positive controls, respectively. Representative normalized autocorrelation curves for Dil‐labeled phosphatidylserine (PS) liposomes g) in the absence (black) and presence (red) of Lycosin‐I and Dil‐labeled PC liposomes h) in the absence (black) and presence (red) of Lycosin‐I, respectively. G (Tau): magnitude of auto‐correlation. It was calculated as described in the Methods section. Values in Figure 2f are presented as mean ± S.E.M.; *n* = 6 for each group.

The interaction between peptides and cancer cell membranes is commonly regarded as the first step of the anticancer efficacy for ACPs, ^[^ ^22^ ^]^ therefore, the binding ability of Lycosin‐I to membranes was explored. Lycosin‐I was conjugated and synthesized with the dye Cy5 (Cy5‐Lycosin‐I). Cell localization experiments showed that 2.5 µm Lycosin‐I (red) could bind to the cell membrane (green) of K562 cells. However, no obvious distribution of red fluorescence was observed in lymphocytes at the same concentration of Cy5‐Lycosin‐I (**Figure** **2**a). 2.5 µm Cy5‐Lycosin‐I can also efficiently bind the U937, HL‐60, and MV4;11 cell lines (Figure S8, Supporting Information). Accordingly, quantitative analysis using Cellometer K2 indicated that 92.31% of K562 cells were labeled with red fluorescence, but only 19.33% of lymphocytes were red‐labeled (Figure 2b). Many studies have shown that membrane lysis following the binding of ACPs to cancer cell membranes is the main mechanism by which ACPs and their analogues induce cell death. Therefore, the membrane lysis activity of Lycosin‐I was investigated. TEM was performed to visualize the morphology of K562 cells after treatment with Lycosin‐I. Compared with the complete cell morphology in the control group, there was obvious leakage of cytoplasmic contents in the cells after treatment with 10 µM Lycosin‐I, indicating damage to the cell membrane (Figure 2c). SYTO9/PI double staining and trypan blue staining were used to assess the permeabilized effect of Lycosin‐I on cell membranes. Compared to the untreated group labeled with green SYTO9, almost all K562 cells were stained red when treated with 10 µm Lycosin‐I for 1 h, as PI penetrated and stained these permeabilized cells; note that this effect was dose‐dependent and almost no red‐labeled cells were observed when Lycosin‐I was treated at low concentrations (≤5 µm) (Figure 2d). Trypan blue staining (a cell‐active dye) also showed that most K562 cells treated with 10 µM Lycosin‐I for 1 h were stained blue (Figure 2e). Moreover, 1 h treatment with Lycosin‐I triggered the release of LDH from K562 cells, especially at high concentrations (>10 µm). These data indicate that the integrity of cell membrane was disrupted because of the membrane lysis activity of Lycosin‐I (Figure 2f), i.e., Lycosin‐I was able to cause direct and rapid destruction of the cell membrane at high concentrations after binding to the K562 cell membranes.

The data described above show that Lycosin‐I has a distinct binding affinity to K562 cells and normal cells. Cancer cells are typically exposed to increased concentrations of negatively charged phospholipids such as PSon the exoplasmic face of the plasma membrane compared to normal cells with neutral phospholipids such as phosphatidylcholine (PC) (Figure S9a, Supporting Information) and sphingomyelin on the outer leaflet of the plasma membrane.^[^ ^22^ ^]^ The electrostatic attraction between negatively charged phospholipids such as PS etc. and cationic ACPs on cell membranes plays an important role in the strong and selective interaction and destruction of cell membranes.^[^ ^23^ ^]^ Then, we investigated whether Lycosin‐I exerts selective anticancer effect by preferentially interacting with PS. PS and PC liposomes were prepared by extrusion, and the competitive assay showed that the addition of PS liposomes, but not PC liposomes, significantly attenuated the inhibitory effect of 10 µm Lycosin‐I (Figure S9b, Supporting Information). This inhibitory activity was confirmed in the U937, HL‐60, and MV4;11 cell lines (Figure S9c–e, Supporting Information). Furthermore, a fluorescence correlation spectrometer (FCS)^[^ ^24^ ^]^ was used to verify the interaction between Lycosin‐I and PS or PC liposomes. PS or PC liposomes were first prepared and labeled with DiL (a lipophilic membrane dye), and the autocorrelation signals in the absence and presence of Lycosin‐I were recorded over time. It was found that treatment with Lycosin‐I resulted in a rightward shift of the autocorrelation curves of PS liposomes (Figure 2g), indicating slower diffusion of the particles due to their larger size, suggesting that the Lycosin‐I molecules were attracted to the surface of PS liposomes and consequently formed larger and stable complexes. This was not the case between Lycosin‐I and PC liposomes (Figure 2h). These data qualitatively suggest that the preferential binding of Lycosin‐I to cancer cell membranes may be a pivotal determinant of its selective effect on leukemia cellsinvolving PS.

### 2.4 Lycosin‐I Induces Apoptosis in K562 Cells

**Figure 3:**
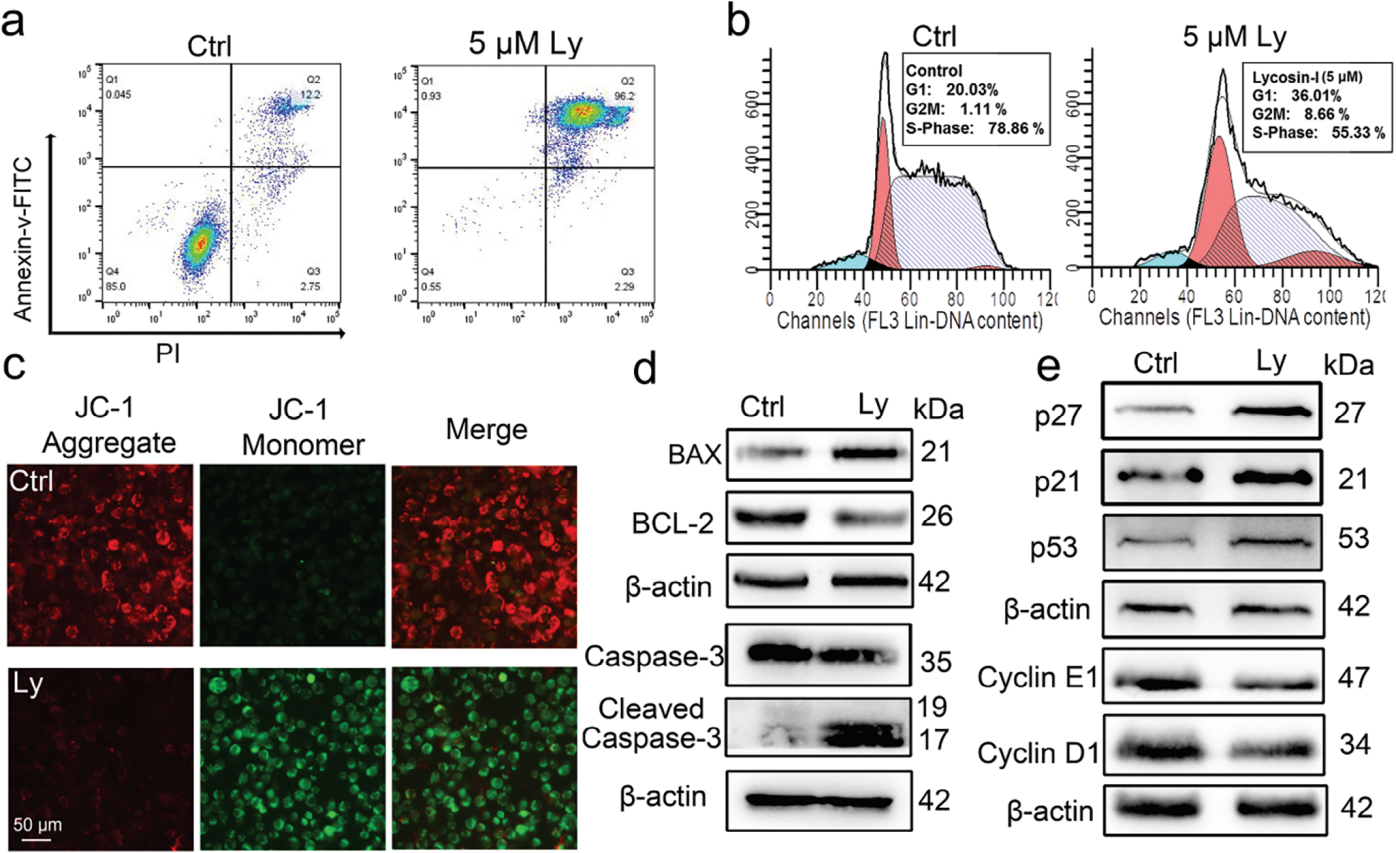
Lycosin‐I induces apoptosis and cell cycle arrest in K562 cells. a) Apoptosis analysis of K562 cells treated with 5 µm Lycosin‐I for 24 h followed by staining with Annexin‐V/PI. b) Cell cycle analysis of K562 cells treated with 5 µm Lycosin‐I for 24 h by flow cytometry. c) Fluorescence images of MMP staining with JC‐1 in K562 cells in the absence or presence of 5 µm Lycosin‐I for 24 h. d,e) The expression of apoptosis‐related proteins BAX, BCL‐2, Caspase‐3, and Cleaved Caspase‐3 (d), and cell cycle‐related proteins p53, p27, p21, cyclin D1, and cyclin E1 (e) in K562 cells treated with 5 µm Lycosin‐I for 24 h. Quantification of proteins (as in Figure S12, Supporting Information) is normalized to β‐actin with respect to the control group (*n* = 3; mean ± S.E.M.) using t‐test, *p < 0.05; **p < 0.01.

Lycosin‐I was able to induce cell death by direct and rapid membrane lysis especially at high concentrations. Note that the IC_50_ value of Lycosin‐I is only 1.66 µm, at which the membrane‐lytic activity was weak, suggesting that other mechanisms should be involved at relatively low concentrations. Therefore, we determined whether Lycosin‐I has the ability to induce cell apoptosis and inhibition of proliferation. Compared with the control, Lycosin‐I increased the percentage of apoptosis in K562 cells in a concentration‐dependent manner (**Figure** **3**a; Figure S10a, Supporting Information). Furthermore, we found that Lycosin‐I led to cell accumulation in the G1 phase and decreased the cell number in the S fraction in a dose‐dependent manner (Figure 3b; Figure S10b,c, Supporting Information), suggesting that Lycosin‐I impedes cell cycle progression and arrests cells in the G1 phase.

Lycosin‐I could induce the decrease of mitochondrial membrane potential (MMP) as shown by the transformation of JC‐1 from red fluorescence in the control group to green fluorescence in the 5 µm Lycosin‐I treatment group (Figure 3c; Figure S11, Supporting Information), indicating the permeabilization of the mitochondrial membrane. BAX and BCL‐2 are involved in the formation of pores in the outer mitochondrial membrane, which triggers the release of cytochrome c into the cytoplasm and the activation of caspase 3, thus playing an important role in mitochondria‐mediated apoptosis.^[^ ^25^, ^26^ ^]^ Western blotting analysis showed that Lycosin‐I significantly increased BAX expression and decreased BCL‐2 expression (Figure 3d; Figure S12, Supporting Information). Increased cleavage of Caspase‐3 was observed in K562 cells treated with 5 µm Lycosin‐I (Figure 3d; Figure S12, Supporting Information), indicating activation of Caspase‐3 and successive execution of apoptosis. These data suggest that Lycosin‐I can induce apoptosis of K562 cells via the mitochondria‐mediated Caspase‐3 pathway.

We also examined the effect of Lycosin‐I on key proteins of the cell cycle. High expression of cyclin‐dependent kinases (CDKs) and cyclins can promote the progression of G0/G1 phase.^[^ ^27^ ^]^ However, p21 and p27, as inhibitors, can downregulate the expression of CDKs, leading to cell cycle arrest in G0/G1 phase, and p53, as an upstream protein of p21 and p27, can positively regulate the progression of this process.^[^ ^27^ ^]^ As expected, administration of 5 µm Lycosin‐I significantly increased the expression of p53, p21 and p27 and downregulated cell cycle regulators cyclin D1 and cyclin E1, which are important for the G1/S transition (Figure 3e; Figure S12, Supporting Information). Meanwhile, the high expression of the p53 protein can promote the occurrence of apoptosis.

### 2.5 Lycosin‐I Causes Ferroptosis in K562 Cells

**Figure 4:**
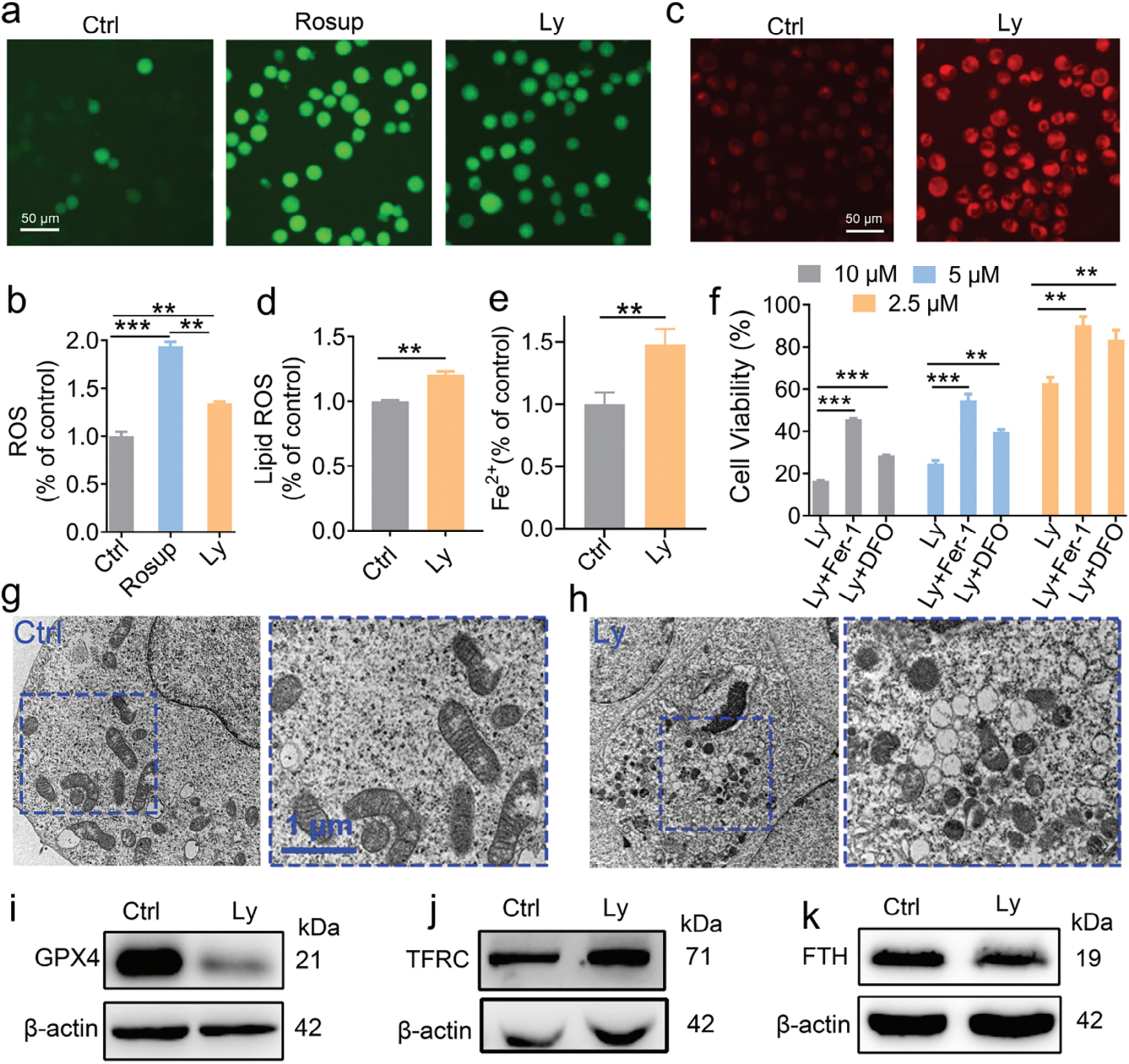
Lycosin‐I induces ferroptosis in K562 cells. a) Representative fluorescence images of ROS levels in K562 cells treated with 5 µM Lycosin‐I for 24 h. Rosup was used as positive control. Scale bar, 50 µm. c) Representative fluorescence images of lipid ROS levels in K562 cells treated with 5 µm Lycosin‐I for 24 h. Scale bar, 50 µm. b–e) Quantitative analysis of ROS contents (b), lipid ROS level (d) and ferrous ion concentration (e) determined using a multifunctional fluorescence microplate reader. f) The cytotoxicity of Lycosin‐I on K562 cells with and without the presence of the ferroptosis inhibitors DFO and Fer‐1. g,h) Representative TEM images of mitochondria in K562 cells. Cells were treated with 5 µm Lycosin‐I for 24 h. i–k) The expression of GPX4 (i), TFRC (j) and FTH protein (k) in K562 cells treated with 5 µm Lycosin‐I for 24 h. Data are presented as mean ± S.E.M., *n* = 6 for each group. Statistical analysis was performed with a t‐test for d and e and with a one‐way ANOVA test for b and f. *p < 0.01; ***p < 0.001. Quantification of proteins (as in the Figure S14, Supporting Information) was normalized to β‐actin relative to the control group (*n* = 3; mean ± S.E.M.) using the t‐test, *p < 0.05; **p < 0.01.

Lycosin‐I can induce membrane perforation, suggesting that Lycosin‐I may induce ferroptosis in cells (Figure S13, Supporting Information). Ferroptosis refers to iron‐dependent regulated cell death (RCD) caused by excessive lipid peroxidation leading to rupture of the plasma membrane.^[^ ^28^ ^]^ Accumulation of ROS, especially lipid ROS, is one of the hallmarks of ferroptosis.^[^ ^29^ ^]^ Indeed, treatment with 5 µm Lycosin‐I significantly promoted the accumulation of total ROS (**Figure** **4**a,b) and lipid ROS (Figure 4c,d). Staining of cells with Ferro Orange (a ferrous ion probe) showed that Lycosin‐I treated cells contained excessive Fe^2+^ (Figure 4e). The two ferroptosis inhibitors DFO and Fer‐1 were able to significantly attenuate the inhibitory effect of Lycosin‐I against K562 cells (Figure 4f). TEM observations showed that K562 cells treated with 5 µM Lycosin‐I for 24 h exhibited the distinctive morphological features of smaller mitochondria with increased membrane density, vacuolization and enlargement of cristae (Figure 4g,h). These data suggest that Lycosin‐I treatment causes ferroptosis in K562 cells.

To clarify the molecular mechanism of Lycosin‐I‐induced ferroptosis, we examined the effects of Lycosin‐I treatment on the expression of some key proteins that regulate ferroptosis. The results showed that the expression of the antioxidant enzyme glutathione peroxidase 4 (GPX4), a key protein that regulates lipid peroxidation, was significantly decreased after treatment with Lycosin‐I (Figure 4i; Figure S14a, Supporting Information). The cysteine‐glutamate antiporter known as the XC‐system also regulates lipid peroxidation. It consists of two proteins, the light chain SLC7A11 and the heavy chain SLC3A2.^[^ ^30^ ^]^ However, there was no significant effect on the expression of SLC7A11 and SLC3A2 at the transcriptional level after Lycosin‐I treatment (Figure S15, Supporting Information). Furthermore, we investigated how Lycosin‐I increased intracellular Fe^2+^ in K562 cells. Iron ions usually bind to transferrin in the form of Fe^3+^, enter the cells through the transferrin receptor TFR1 (TFRC) and are then reduced to Fe^2+^ by the metal reductase STEAP3. The Fe^2+^ in the cells can combine with ferritin to form an iron pool for storage. Our Western blotting analysis showed that Lycosin‐I treatment could increase the expression of TFRC (Figure 4j; Figure S14b, Supporting Information) and decrease the expression of ferritin heavy chain (FtH) (Figure 4k; Figure S14c, Supporting Information). Ferritin consists of a ferritin heavy chain (FTH) and a ferritin light chain (FTL), the degradation of which, known as ferritinophagy, increases intracellular iron oxygen and free radicals to promote ferroptosis.^[^ ^31^ ^]^ These data suggest that Lycosin‐I may enhance Fe^3+^ transport into cells through increasing TFRC expression and improve Fe^2+^ release from the iron pool by inducing ferritinophagy, ultimately leading to increased intracellular Fe^2+^ concentration.

### 2.6 Lycosin‐I Suppresses the PI3K‐AKT‐mTOR Signaling Pathway to Induce Apoptosis, Cell Cycle Arrest, and Ferroptosis in K562 Cells

**Figure 5:**
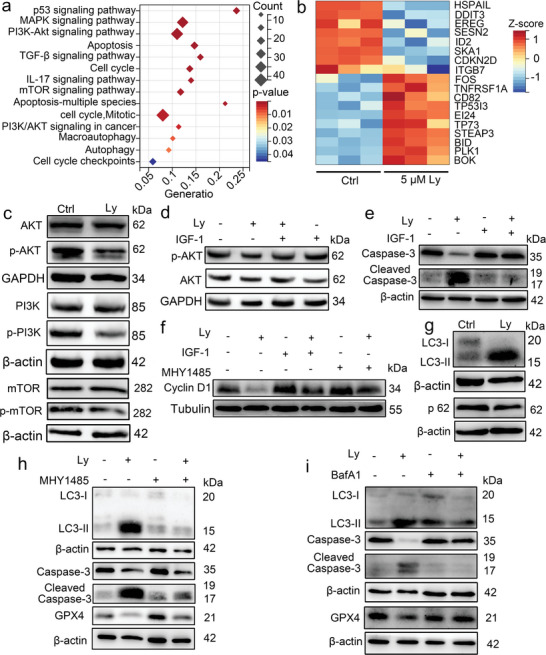
Lycosin‐I triggers apoptosis, autophagy, cell cycle arrest, and ferroptosis of K562 cells via the PI3K‐AKT‐mTOR signaling pathway. a) The KEGG signaling pathway enrichment analysis based on the differentially expressed genes in K562 cells treated with 5 µm Lycosin‐I. b) Cluster heatmap of the expression differences of genes regulating cell survival‐related signaling pathways as mentioned in Figure 5a. c–i) Western blotting analysis of the proteins indicated in K562 cells treated with 5 µm Lycosin‐I and some pharmacological agents for 24 h. c) Lycosin‐I decreases the phosphorylation of PI3K, AKT and mTOR proteins. d,e) The AKT activator IGF (200 ng mL^−1^) reverses the inhibition of AKT phosphorylation d) and the cleavage of Caspase‐3 (e) by Lycosin‐I. f) The mTOR activator MHY1485 (1 µm) or IGF (200 ng mL^−1^) reverses the down‐regulation of Cyclin D1 by Lycosin‐I. g) Lycosin‐I improves the ratio of LC3‐II to LC3‐I proteins and decreases the expression of p62. h,i) MHY1485 (1 µm) (h) or the autophagy inhibitor BafA1(40 nm) (i) suppresses Lycosin‐I‐induced autophagy and prevents apoptosis and ferroptosis. Quantification of proteins (as shown in the Figures S18 and S19, Supporting Information) is normalized to the internal control with respect to the control group (*n* = 3; mean ± S.E.M) using t‐test or one‐way ANOVA test; *p < 0.05; **p < 0.01; ***p < 0.01.

To better understand the molecular mechanisms of Lycosin‐I, transcriptomic analysis was performed (Figure S16a,b, Supporting Information). KEGG signaling pathways such as PI3K‐AKT signaling, mTOR signaling, apoptosis, and cell cycle, etc., all of which play important roles in cell survival and growth, were enriched after 5 µm Lycosin‐I treatment (**Figure** **5**a). In addition, core genes involved in the above signaling pathways such as *HSPAIL*, *DDIT3*, *EREG*, *SESN2*, *ID2*, *SKA1*, and *CDKN2D* were significantly downregulated and *TNFRSF1A*, *TP5313*, *TP73*, *BID*, and *BOK*, etc., were markedly upregulated after treatment with Lycosin‐I (Figure 5b). The PPI and network generated by some of the differentially expressed genes in the signaling pathways shown in Figure 5a suggest that these pathways are closely interacted with each other, and that the PI3K‐AKT‐mTOR signaling pathway may play a cental role in the effect of Lycosin‐I on K562 cells (Figure S17, Supporting Information). We therefore examined the expression of proteins related to this signaling pathway in K562 cells treated with Lycosin‐I.

We found that the expression of phosphorylated PI3K, AKT and mTOR proteins was downregulated in the Lycosin‐I group, although the expression of AKT, PI3K, and mTOR themselves was not altered (Figure 5c; Figure S18, Supporting Information), suggesting that Lycosin‐I was able to reduce the activation of PI3K, AKT, and mTOR. In addition, Lycosin‐I was able to partially antagonize the effect of IGF‐1 on p‐AKT activation (Figure 5d; Figure S19a, Supporting Information), providing further evidence that Lycosin‐I regulates the PI3K‐AKT‐mTOR pathway. This signaling pathway mainly modulates intracellular signal transduction and various biological processes such as cell cycle, apoptosis, and inflammation, etc.^[^ ^26^, ^32^ ^]^ Therefore, we were interested in whether this signaling pathway could be involved in the regulation of Lycosin‐I‐induced apoptosis, cell cycle arrest, and even ferroptosis. Our results show that IGF‐1 decreased the cleavage of Caspase‐3 in K562 cells treated with 5 µm Lycosin‐I, (Figure 5e; Figure S19b, Supporting Information), and IGF‐1 and the mTOR activator MHY1485 also rescued the downregulation of cyclin D1 protein (Figure 5f; Figure S19c, Supporting Information), supporting that Lycosin‐I could induce apoptosis and cell cycle arrest in K562 cells by suppressing the PI3K‐AKT‐mTOR signaling pathway.

Autophagy‐related signaling pathways were also enriched in K562 cells after Lycosin‐I treatment (Figure 5a), and the mTOR signaling pathway was extensively involved in the regulation of autophagy.^[^ ^33^ ^]^ Western blotting analysis showed that Lycosin‐I significantly improved the ratio of LC3‐II to LC3‐I proteins and decreased the expression of p62, marker of autophagic flux in K562 cells (Figure 5g; Figure S18, Supporting Information), and this effect could be reversed by treatment with the mTOR activator MHY1485 (Figure 5h; Figure S19d, Supporting Information), suggesting that autophagy in K562 cells treated with Lycosin‐I may act as a downstream part of the PI3K‐AKT‐mTOR signaling pathway. This also attenuated Lycosin‐I‐induced apoptosis and ferroptosis, as shown by treatment with MHY1485, which inhibited cleavage of Caspase 3 and downregulation of GPX4 induced by Lycosin‐I (Figure 5h; Figure S19e,f, Supporting Information). And Lycosin‐I‐induced autophagy was directly inhibited by the autophagy inhibitor BafA1 (Figure 5i; Figure S19g–i, Supporting Information). Furthermore, the cell viability assay of Lycosin‐I was performed using the inhibitors of apoptosis (Z‐VAD‐FMK), ferroptosis (Fer‐1), and autophagy (BafA1) to clarify the relationship among autophagy, apoptosis, and ferroptosis. As shown in Figure S20a (Supporting Information), Lycosin‐I‐induced autophagy could indeed cause cell death through regulating apoptosis and ferroptosis, but we cannot exclude other mechanisms of Lycosin‐I‐induced apoptosis and ferroptosis. Moreover, even the co‐treatment with all three inhibitors cannot completely rescue Lycosin‐I‐treated K562 cells compared with the control group, because direct cell membrane lysis is also important for Lycosin‐I‐induced cell death. These results were also confirmed by the cell viability assay using by the flow cytometer (Figure S20b, Supporting Information). Taken together, these results suggest that autophagy may play a role in the regulation of apoptosis and ferroptosis upon Lycosin‐I treatment.

### 2.7 Evaluation of the Anticancer Activity and Safety of Lycosin‐I In Vivo

**Figure 6:**
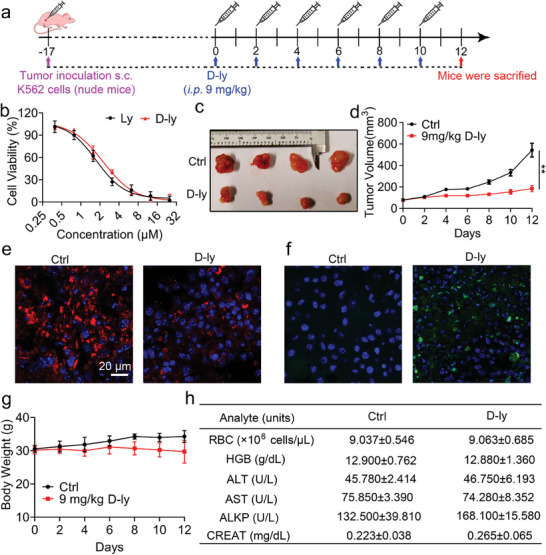
In vivo antitumor activity and safety evaluation of Lycosin‐I. a) Schematic illustration of the schedule for the in vivo therapeutic experiments. Treatments were administered via intraperitoneal injection every other day for a total of 6 times. b) Cell viability of Lycosin‐I and D‐Lycosin‐I on K562 cell lines was examined using the CCK‐8 assay. Cells were incubated with various concentrations of the peptides for 24 h. c) Growth curves of K562 tumor formation. d) Images of excised K562 tumors in nude mice (*n* = 4). e) Analysis of GPX4 expression in tumor of control and D‐Lycosin‐I treatment groups. f) Analysis of cleaved‐Caspase‐3 expression in the tumor of the control and D‐Lycosin‐I treatment groups. g) Changes in body weight of BALB/c mice during the study. h) Data from RBC parameters, and liver and kidney function tests, of mice treated with PBS or D‐Lycosin‐I for 12 days. HGB, hemoglobin; ALT, alanine aminotransferase; AST, aspartate aminotransferase; ALKP, alkaline phosphatase; Creat, creatinine. Data are expressed as mean ± S.E.M., *n* = 4 for each group. Statistical analysis was performed using the t‐test. *p < 0.01; **p < 0.01.

Inspired by the cytotoxicity of Lycosin‐I in the in vitro cell lines and clinically isolated cells, we investigated the antitumor efficacy of Lycosin‐I in vivo. Considering that Lycosin‐I is readily degraded by proteases in vivo, we synthesized the D‐enantiomer of Lycosin‐I with complete D‐amino acid replacement,^[^ ^34^ ^]^ called D‐Lycosin‐I for animal experiments (Figures S21 and S22, Supporting Information). D‐Lycosin‐I had the same activity against K562 as Lycosin‐I (**Figure** **6**b). The treatment schedule is shown in Figure 6a. Approximately 13 days after inoculation (K562 tumor volumes reached 80 to 150 mm^3^), tumor‐bearing female nude mice were divided into two groups and injected intraperitoneally with PBS and 9 mg kg^−1^ D‐Lycosin‐I (200 µL) every other day for a total of 6 times. As shown by the growth curves of K562 tumors in the mice, the tumors in the PBS treatment group continued to grow rapidly, while the tumors in the D‐Lycosin‐I‐treated mice showed no obvious increase in volume during the course of treatment (Figure 6c). The tumors in the latter group were much smaller than those in the former when the tumor tissues were removed (Figure 6d). Both groups showed similar weight gain over the course of treatment (Figure S23, Supporting Information). Moreover, the results of immunofluorescence sections of tumors showed that D‐Lycosin‐I could downregulate the expression of GPX4 (Figure 6e) and upregulate the expression of Cleaved‐Caspase‐3 (Figure 6f), suggesting that D‐Lycosin‐I could induce ferroptosis and apoptosis of tumor cells in vivo.

Subsequently, we conducted a systematic study to evaluate the safety of D‐Lycosin‐I in vivo. BALB/c mice were divided into two groups and received a total of 6 intraperitoneal injections of PBS and 9 mg kg^−1^ D‐Lycosin‐I (200 µL) every other day (Figure S24a, Supporting Information). We observed no changes in body weight between the mice of the two groups (Figure 6g). In addition, blood and tissues were collected for RBC function parameters, liver and kidney function, and H&E staining. More importantly, in the animals injected with D‐Lycosin‐I injection, RBC, liver and kidney parameters remained within the normal range (Figure 6h). Furthermore, H&E staining of heart, liver, spleen, lung kidney sections showed that D‐Lycosin‐I caused no obvious damage to these organs in the mice compared with the control (Figure S24b, Supporting Information). Taken together, these data suggest that D‐Lycosin‐I can effectively inhibit tumor growth in vivo without significant signs of toxicity, and this good safety may be due to its low hemolytic activity and low toxicity to normal cells (Figure 1).

## 3 Discussion

Although conventional chemotherapy plays a crucial role in the clinical treatment of leukemia, it is accompanied by serious problems such as toxicity and drug resistance. ACPs represent a promising alternative to conventional chemotherapy. Spider venoms containing multiple ACPs have demonstrated the ability to kill tumor cells or reduce their proliferation without developing resistance.^[^ ^35^, ^36^ ^]^ In previous studies, we have reported that a peptide, Lycosin‐I, isolated from spider venom, exhibits significant anticancer activity.^[^ ^18^ ^]^ The characteristics of high potency and selectivity confer advantages to Lycosin‐I with in the development of effective drugs against leukemia. Indeed, in vivo studies have shown that Lycosin‐I is highly effective in inhibiting tumor growth while exhibiting good safety in the K562 xenograft mouse model. Our study has also shown that the potent and selective cytotoxicity of Lycosin‐I on leukemia cells may be due to the mechanism involving preferential binding to cancer cell membranes, followed by rapid membrane lysis, cell apoptosis, cell cycle arrest and ferroptosis.

Lycosin‐I undergoes a conformational transformation from a random coil to a helix upon binding to lipids, which enables it to target cell membranes and induce membrane lysis, leading to permeation or destruction of the cancer cell membrane. In fact, membrane lysis leading to cell death is the main mechanism of action for most anticancer peptides.^[^ ^37^ ^]^ Our study demonstrated that Lycosin‐I has high cytotoxic activity against K562 cells and can induce membrane lysis in these cells as shown by LDH release, scanning electron microscopy (SEM) analysis, and AO/EB assay, suggesting that Lycosin‐I binds to the cell membrane and induces cell death. D‐Lycosin‐I containing D‐amino acid substitutions showed the same cytotoxic activity as Lycosin‐I with L‐amino acid residues. This suggests that it is less likely that Lycosin‐I directly interacts with protein targets with specific spatial conformations. In addition, our previous study has shown that Lycosin‐I anchors onto the lipid membrane and forms aggregates and oligomers^[^ ^38^ ^]^ reducing the diffusion dynamics after adsorption to the lipid membrane and altering membrane conformation. We therefore speculate that the direct target of Lycosin‐I is the lipids in the cell membrane.

**Scheme 1:**
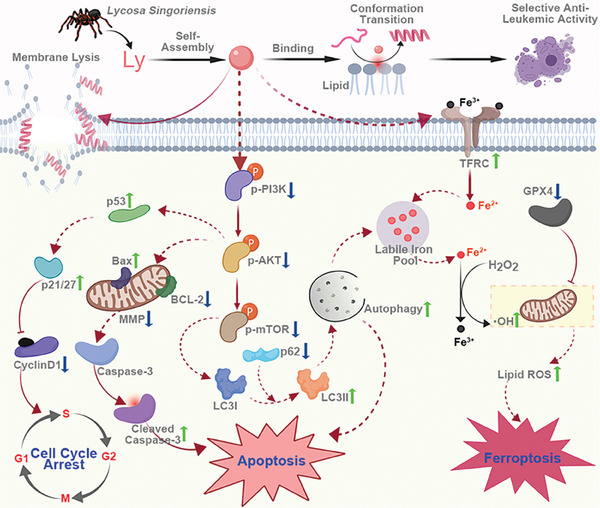
Illustrates the multimodal mechanism of the lipid‐sensitive peptide Lycosin‐I, which acts selectively against leukemia. For the sake of simplicity, Lycosin‐I is abbreviated as Ly in all figures in this study. Lycosin‐I, which was isolated from the venom of *Lycosa singorensis*, has the ability to self‐assemble into nanosphere structures. Upon binding to phospholipids, Lycosin‐I undergoes a conformational change from a random coil to a helix, resulting in a potent inhibitory effect on leukemia cells via a multimodal mechanism. 1) Rapid membrane lysis at high concentration. 2) Induction of cell apoptosis: Lycosin‐I activates the mitochondria‐mediated apoptotic pathway, which ultimately leads to Caspase‐3 cleavage and cell apoptosis. 3) Induction of cell cycle arrest in G1 phase: Lycosin‐I upregulates the expression of p53, p27, and p21 while inhibits cyclin D and E, leading to cell cycle arrest in the G1 phase. 4) Induction of ferroptosis of cells: Lycosin‐I increases the expression of transferrin, enhances intracellular Fe^2+^ levels, and decreases the expression of GPX4, leading to disruption of iron homeostasis and lipid peroxidation,eventually inducing ferroptosis. Of course, ferroptosis also can ultimatelycause the cell membrane lysis. 5) Activation of cell autophagy: Lycosin‐l increases the LC3‐II/LC3‐I ratio and reduces the expression of p62, leading to activation of autophagy and further promoting cell apoptosis and ferroptosis. Most importantly, apoptosis, cell cycle arrest, and ferroptosis appear to be regulated by the Pl3K‐AKT‐mTOR signaling pathway. The graphics of Scheme 1 and ToC were created and licensed by MedPeer (medpeer.cn).

Charge attraction between ACPs and cell membranes is a crucial step in inducing cell membrane lysis, apoptosis and inhibition of proliferation. The outer leaflet of normal mammalian cell membranes is mainly composed of neutral amphipathic phospholipids, whereas cancer cell membranes exclusively consist of anionic components such as phosphatidylserine (PS), heparin sulfate, and sialylated ganglioside.^[^ ^22^, ^23^ ^]^ Some anticancer peptides, such as D‐K6L9, lactoferricin and temporin‐1CEa exhibit selective anticancer activity by specifically binding to phosphatidylserine on the surface of cancer cells.^[^ ^39^, ^40^, ^41^ ^]^ In this study, it was found that a significant amount of Cy5‐labeled Lycosin‐I was enriched on the surface of K562 cells, but hardly bound to normal lymphocytes. This suggests that the selective anticancer effect of Lycosin‐I is determined by the specific binding of Lycosin‐I to leukemia cell membranes. Our FCS assay also showed that Lycosin‐I can preferentially bind to PS. Therefore, the strong interaction between Lycosin‐I and K562 cells may be due to the increased PS content in the K562 cell membrane, whereby Lycosin‐I selectively inhibits leukemia cells and has weak activity on non‐cancerous cells and normal human lymphocytes. After selective attack oncell membranes, Lycosin‐I can inhibit cell growth via various pathways (Scheme 1). At high concentrations (e.g., 10 µm), Lycosin‐I can rapidly destroy cell membranes (e.g., within 1 h), leading to cell death. At relatively low concentrations (e.g., 5 µm), it can induce ferroptosis and apoptosis and arrest the cell cycle.

In recent years, it has been documented that the PI3K‐AKT‐mTOR signaling pathway is highly active in CML, and its abnormal activation contributes to multidrug resistance in CML.^[^ ^42^, ^43^, ^44^ ^]^ Our results show that Lycosin‐I induces apoptosis, cell cycle arrest and ferroptosis in K562 cells by suppressing the PI3K‐AKT‐mTOR signaling pathway. Extensive studies suggest that this signaling pathway is closely associated with apoptosis‐related factors, including the BCL‐2 protein family, and cell cycle regulators such as cyclins and cyclin‐dependent kinase inhibitors (CDKIs).^[^ ^45^, ^46^ ^]^ For example, phosphorylation of AKT directly promotes the phosphorylation of Bad protein (p‐Bad), which negatively regulates the formation of the pro‐apoptotic complex, and then promotes the expression of the anti‐apoptotic factors BCL‐2 or Bcl‐xL, thereby inhibiting cell apoptosis.^[^ ^45^ ^]^ It is now well established that PI3K‐AKT‐mTOR^[^ ^46^ ^]^ and MAPK/mTOR^[^ ^47^ ^]^ activate autophagy. In this study, we demonstrated that Lycosin‐I can significantly induce autophagy in K562 cells, and further promote apoptosis and ferroptosis. Cell cycle progression is precisely controlled by cell cycle activators such as cyclin D1 and cyclin‐dependent kinases (CDK) and cell cycle inhibitors such as the cyclin‐dependent kinase inhibitors p21 and p27.^[^ ^48^ ^]^ Our study showed that the PI3K‐AKT‐mTOR signaling pathway regulates the expression of cell cycle activators such as cyclin D1, leading to the downregulation of cyclin D1 expression and cell cycle arrest in G1/S phase (Scheme 1) after treatment with Lycosin‐I. More interestingly, Lycosin‐I can induce ferritinophagy by inhibiting the mTOR signaling pathway and increase the expression of transferrin and decrease the expression of GPX4, which led to disorder of iron homeostasis and lipid peroxidation, and eventually induced ferroptosis in K562 cells (Scheme 1).

Ferroptosis is driven by iron‐dependent phospholipid peroxidation.^[^ ^28^ ^]^ The ferroptosis inducer has shown promise as a potential cancer therapy, either as a single agent or in combination with other targeted agents, including PI3K/mTOR pathway inhibitors and immune checkpoint blockers.^[^ ^49^ ^]^ Lycosin‐I‐induced ferroptosis in leukemia provides an effective basis for the use of Lycosin‐I in combination therapy. In addition, cancer cells often develop resistance to apoptosis, a common form of programmed cell death. Ferroptosis offers another way to induce cell death, making it a potential strategy to overcome the resistance mechanisms observed in certain types of cancers. For example, induction of ferroptosis and necroptosis contributed to erastin‐induced growth inhibition and overcame drug resistance in AML cells.^[^ ^50^ ^]^ However, the interplay between the different types of cell death induction mentioned in our study and their regulatory network remains to be further elucidated. In summary, the selective anti‐leukemia activity of Lycosin‐I is orchestrated by a combination of self‐assembly, conformational changes, selective membrane binding, membrane lysis, apoptosis, cell cycle arrest, and ferroptosis induction. This multimodal mechanism positions Lycosin‐I as a promising candidate for the development of effective and selective drugs against leukemia.

## 4 Experimental Section

### Chemical Synthesis of Peptides

The peptides Lycosin‐I (KGWFKAMKSIAKFIAKEKLKEHL), D‐Lycosin‐I (kgwfkamksiakfiakeklkehl; lowercase letters denote D‐amino acids.), and cy5‐Lycosin‐I were synthesized on a Rink amide‐AM resin using the SPPS (solid‐phase peptide synthesis) method as previously described.^[^ ^19^ ^]^

### Cell Lines and Cell Culture

All cell lines were obtained from National Collection of Authenticated Cell Cultures or saved in the lab and cultured in RPMI‐1640 medium or DMEM medium or F12K medium supplemented with 10% (v/v) fetal bovine serum (FBS), 100 units mL^−1^ penicillin, 100 µg mL^−1^ streptomycin, and 0.05% (v/v) L‐glutamine serum in a humidified atmosphere of 5% CO_2_ at 37 °C.

### Patient Samples and Lymphocyte Separation

Patient samples including fresh primary bone marrow (BM) and peripheral blood (PB) from leukemia patients (Table 1) and peripheral blood from healthy individuals were tested. The subsets of leukemia were diagnosed according to standard clinical and laboratory criteria. All samples were obtained from Second XiangYa Hospital, Central South University, Changsha, China. Written informed consent was obtained from all patients in accordance with the Hospital Ethical Committee's requirements. Lymphocytes were isolated according to the instructions of the Human Lymphocyte Separation Medium (Cat. No.: DKW‐KLST‐015, Dakewe).

### Cell Viability Assays

To determine the effects of Lycosin‐I on cell cytotoxicity, the CCK8 assay was performed according to the manufacturer's protocol. Cells and primary patient cells were plated in 96‐well plates and treated with Lycosin‐I at a range of concentrations for 24 h. Subsequently, these cells were incubated with CCK8 (Cat#C0038, Beyotime) for 1–4 h and the absorbance at 450 nm was measured using a Microplate Reader (BioTek, USA). The percentage of cellular cytotoxicity was calculated using the following equation: Cell viability (%) = (OD_sample_‐ OD_blank_) / (OD_control_ – OD_blank_) ×100%. IC_50_ values were calculated using GraphPad Prism 9. The abscismal in the fitted curve was the concentration treated with the peptide instead of the logarithm of the concentration after fitting.

### Atomic Force Microscopy (AFM)

AFM experiments were performed with a Bruker ICON instrument under the tapping mode. The Lycosin‐I stock solution (10 µL, 100 µm) was pipetted onto the cleaned mouse surface and the liquid retained on the mica surface was removed with filter paper. The mica slides were dried under atmospheric conditions prior to the AFM experiments.

### Characterization of the Secondary Structure

The secondary structure of Lycosin‐I in different solutions was determined with the Jasco‐810 spectropolarimeter using 2 mm quartz slides. All spectra were recorded with a wavelength interval of 0.5 nm. The final concentration of the peptide samples was 50 µm.

### Fluorescence Staining Analysis

K562 cells and isolated lymphocytes (≈1 × 10^5^ cells mL^−1^) were resuspended in serum‐free 1640 medium and then plated in six‐well plates with sterile coverslips pre‐coated with poly‐l‐lysine (1 mg mL^−1^), to promote cell adhesion to the coverslips. The adherent cells were treated with 2.5 µm cy5‐Lycosin‐I for 1 h. The cells on the coverslips were then fixed with paraformaldehyde (4% (w/v) in PBS). The cells were then washed extensively in PBS and exposed to DAPI and Dio (1:1000) for 45 min. Images were captured using a confocal laser‐scanning microscope (LSM 510, Carl Zeiss). In addition, the number of fluorescence‐labeled cells was measured with the Cellometer K2.

### The Cytotoxicity of Lycosin‐I When Incubated with POPS and POPC Liposomes

The liposomes were prepared according to the methods described in the literature.^[^ ^51^ ^]^ The POPS and POPC powder were dissolved in chloroform and the chloroform was removed by rotation. The resulting POPS and POPC lipid film was dissolved with 1 mL of 50 mm HEPES‐KOH (pH 7.6) by sonicating evaporator at 37 °C. Subsequently, the lipid solution was processed stepwise with polycarbonate membranes with a pore size of 100 nm through a micro‐extruder (Avanti Polar Lipids, America). For the cytotoxicity assay, after pretreatment of the cells with POPS or POPC lipid in serum‐free 1640 medium for 2 h, Lycosin‐I (final concentration 10 µm) was added to the 1640 medium for 1 h, and cell viability was determined with CCK‐8.

### FCS Experiments and Data Analysis

The binding ability between Lycosin‐I and POPS/POPC liposomes was measured using a benchtop FCS instrument (LightEdge Technologies Ltd., Zhongshan, China).^[^ ^52^, ^53^ ^]^ For this purpose, a POPS or POPC liposome labeled with the membrane dye DiL was prepared. The FCS instrument was equipped with two cw‐lasers (488 and 561 nm) and an Olympus 60X NA1.2 water immersion objective. High‐precision coverslips (No. 1.5H, Deckglaser, Sigma Aldrich) were directly equilibrated on top of the Olympus water immersion objective, upon which the Alexa, POPS, or POPC liposome and 10 µm Ly‐liposome were added. The instrument was calibrated with a 10 nm solution of Alexa 561‐carboxylic acid. Subsequently, 100 FCS measurements were performed using the 561 nm laser with each experiment lasting 2 s. Three more sets of such 100 FCS measurements were performed with two new coverslips. The Auto‐correlation curves were analyzed using Correlation Analysis software (LgihtEdge Technologies Ltd.) and the following mathematical model:

(1) $$G\left(\tau \right)=\frac{1-T+T\cdot e^{-\frac{\tau }{\tau_{T}}}}{1-T}\cdot \frac{1}{N}\cdot \frac{1}{1+\frac{\tau }{\tau_{D}}}\cdot \frac{1}{\sqrt{1+\frac{\tau }{\tau_{D}\cdot S^{2}}}}$$

*N* is the number of fluorescently labeled sample molecules in the FCS volume, *τD* is the characteristic diffusion correlation time of the sample molecule, *S* is the structure parameter, *T* is the fraction of fluorophores residing in the triplet state, and *τT* is the triplet lifetime.

### Transmission Electron Microscopy (TEM)

TEM images of Lycosin‐I were acquired using a Tecnai G2F20 microscope with an accelerating voltage of 100 kV. Five microliters of the diluted stock solution (100 µm) was pipetted onto the surface of a carbon‐coated copper grid for 5 min and removed with filter paper. After drying in air, 5 µL of the uranyl acetate solution (2 wt.%) was then pipetted onto the grid subsequently. After being stained for 5 min, the droplet was removed with filter paper. The grids were dried in a desiccator prior to measurement. The internal structure of the cells was analyzed using TEM. Briefly, cells were treated with 5 µm Lycosin‐I for 24 h or 10 µm Lycosin‐I for 1 h and then washed three times with PBS. After fixation with 2.0% glutaraldehyde, negative staining, and dehydration, the cells were embedded in the resin. Then the sections were prepared and transferred to the copper grid. Finally, the samples were analyzed with the TEM.

### Scanning Electron Microscopy (SEM)

K562 cells (2 × 10^5^ cells per well) were resuspended in 1640 serum‐free medium and then plated in 24‐well plates with sterile round coverslips and incubated for 8 h to promote cell adhesion. Cells on the coverslips were cultured in the presence or absence of 5 µm Lycosin‐I for 24 h. Cells were carefully washed with 0.1 m sodium cacodylate and fixed with glutaraldehyde (2.5% (v/v) in sodium cacodylate) for 2 h. The cells were then gently washed again and fixed with osmium tetroxide (1% (w/v) in sodium cacodylate) for 30 min, washed again, and dehydrated in increasing concentrations of ethanol until a final concentration of 100% ethanol was reached. Samples were then dried using a Polaron E3000 Critical Point Dryer (Quorum Technologies, Guelph, ON, Canada). Samples were then mounted onto stubs and coated with gold using the Polaron SC7620 Mini Sputter Coater (Quorum Technologies). The cells were then observed using a Hitachi S4700 scanning electron microscope at ×10000 magnification.

### SYTO/PI Double Staining

K562 cells (≈2 × 10^4^ cells per well) were seeded in a 24‐well plate and treated with 2.5, 5, or 10 µm Lycosin‐I for 1 h. Cells were then stained with 200 µL of a dye mixture containing 0.4 µL SYTO and 0.4 µL PI. After staining, the fluorescence was immediately visualized with a fluorescence microscope.

### LDH Leakage Assay

According to the manufacturer's instructions, the LDH release assay was used to determine the integrity of the membrane. In brief, after the K562 cells were treated with different concentrations of Lycosin‐I for 1 h, the reaction mixture (50 µL) was added to each well and allowed to react at room temperature for 30 min, followed by 50 µL of the stop solution. No peptides were added, indicating that there was no leakage. The cell treated with 1% Triton X‐100 represented 100% leakage (total LDH release). Fluorescence was measured using a microplate reader at 490 nm.

### RNA‐seq Analysis

K562 cells were treated with 5 µm Lycosin‐I in the absence of serum for 24 h. Total cell RNA was extracted using the mirVana miRNA isolation Kit (ambion). The quality and quantity of RNA was assessed using the NanoDrop 2000/2000c (Thermo Scientific,Wilmington, DE, USA). The RNA integrity number (RIN) was analyzed using the Agilent 2100 Bioanalyzer (Agilent Technologies, Santa Clara, CA, USA). The RiboZero rRNA removal kit (Epicentre, USA) for gram‐positive was used to eliminate ribosomal RNA prior to RNA sequencing analysis. Strand‐specific cDNA libraries were generated according to standard procedures for subsequent Illumina sequencing using the Illumina Nova 6000 platform (Shanghai Oebiotech Co., Ltd., Shanghai, China), and 150 bp double‐ended reads were generated. The raw data sequencing reads were processed with Trimmomatic. The cleaned reads were mapped to the human reference genome using Rockhooper 2. The FPKM value of each transcript was calculated with Rockhooper 2. Differentially expressed transcripts were identified using the DESeq R package.^[^ ^54^, ^55^ ^]^

### Flow Cytometric Analysis of Apoptosis and Cell Cycle

K562 cells were analyzed using the Annexin V‐FITC/PI apoptosis kit according to the manufacturer's instructions (BD Biosciences, USA). K562 cells were treated with Lycosin‐I (2.5 and 5 µm) or different inhibitor for 24 h, no drug treatment was designated as the control. K562 cells were harvested and stained with 5 µL Annexin V–FITC, 5 µL PI and incubation buffer. The K562 cells were then subjected to flow cytometryafter an incubation period of 15 min at 37 °C. The percentage of viable (Annexin‐V−/PI−), early apoptotic (Annexin‐V+/PI−), late apoptotic (Annexin‐V+/PI+) and necrotic (Annexin‐V−/PI +) cells was analyzed. For the cell cycle assay, K562 cells were treated with Lycosin‐I (2.5 and 5 µm) for 24 h. After incubation, a total of 3 × 10^5^ cells mL^−1^ were harvested from the treated and normal samples. The K562 cells were washed with PBS and fixed in ice‐cold 70% ethanol. The plates were finally stained with PI (propidium iodide, 1 mg mL^−1^), TritonX‐100 (0.1%), and RNAse (100 mg mL^−1^) and incubated at 37 °C for 20 min in the dark and then analyzed for DNA content using a flow cytometer. Samples were prepared in triplicate with at least three replicates for each experiment. To exclude cell debris and cell clumps from the analysis, appropriate gating was employed. Cell cycle distribution was then determined by flow cytometry (Beckman Coulter, Fullerton, California, USA). Cell cycle phases were determined by recording the peak area of FL3‐A on a linear scale.

### Detection of Mitochondrial Membrane Potential

Mitochondrial membrane potential was measured using the Mitochondrial Membrane Potential detection kit purchased from Shanghai Beyotime Company (C2003S). K562 cells (1 × 10^4^ cells per well) and primary patient cells were plated in 96‐well plates and treated with 5 µm Lycosin‐I for 24 h. Fluorescence intensities (488 nm excitation/525 nm emission; 540 nm excitation/590 nm emission) were then measured using multifunctional microplate reader.

### Determination of ROS Production

ROS levels in the cells were measured using 2ʹ,7ʹ‐dichlorofluore scin diacetate (DCFH‐DA), which was purchased from Shanghai Beyotime Company (S0033S). After treatment with 5 µm Lycosin‐I for 24 h, K562 cells were incubated in 96‐well plates with DCFH‐DA for 30 min at 37 °C. Cells were then measured using a multifunctional microplate reader (488 nm excitation/525 nm emission).

### Lipid Peroxidation

Lipid peroxidation was measured using C11‐BODIPY (Thermo Fisher Scientific). As lipid peroxidation increases, the fluorescence shifts from red to green fluorescence emission. K562 cells in 96‐well plates were incubated with 5 µm Lycosin‐I for 24 h. C11‐BODIPY (581/591) (2.5 µm) was added and incubated for 30 min. The cells were then measured using a multifunctional microplate reader.

### Iron Assay

The intracellular Fe^2+^ was assessed using FerroOrange (Fe^2+^ indicator), which was purchased from DOJINDO company (F374). K562 cells in 96 well plates were incubated with 5 µm Lycosin‐I for 24 h. After treatment, the cells were incubated with FerroOrange for 15 min. The cells were then measured using a multifunctional microplate reader.

### Western Blot Analysis

Whole‐cell extracts were prepared in RIPA lysis buffer (Cat#PL001, Sangon), and protein concentration was measured using the BCA Protein Assay Kit (SK3021, Sangon). Equivalent total proteins from different samples were electrophoresed through 8–12% sodium dodecyl sulfate‐polyacrylamide gels, and the proteins were then electrotransferred onto a PVDF membrane. The following antibodies were used: AKT (10176‐2‐AP), phospho‐AKT (28731‐1‐AP), mTOR (66888‐1‐Ig), phospho‐mTOR (67778‐1‐Ig), p21 (10355‐1‐AP), p27 (25614‐1‐AP), FTH (10727‐1‐AP), Cyclin E1(11554‐1‐AP), p53 (60283‐2‐Ig), LC3 (14600‐1‐AP), p62 (66184‐1‐Ig) were purchased from Proteintech, China. GPX4 (381958), Bax (240117), Bcl‐2 (240122), and Cyclin D1(380999) and PI3K (251221) were purchased from Zenbio, China, and p‐PI3K(AF00823) was purchased from AiFang biological company, China. anti‐Caspase‐3 (Abcam) was purchased from Tianjin Nanzi Trading Co., Ltd. The membranes were incubated with the above primary antibodies overnight at 4 °C and with horseradish peroxidase (HRP)‐conjugated secondary antibodies for 1 h at room temperature.

### Xenograft Using Nude Mice

Healthy male BALB/c nu/nu mice (4 to 6 weeks old) were purchased from Slac & Jingda Corporation of laboratory animals, Changsha, China. All animal experiments were approved by the Institutional Animal Care and Use Committee (IACUC) of Hunan Normal University (approval number: HUNNU2022‐179), and the National Institutes of Health guidelines for the performance of animal experiments were followed. K562 cells (5 × 10^6^ in 100 µL PBS) were injected subcutaneously (s.c.) into the right dorsal of mice. When the tumors reached a size of 80−150 mm^3^, all mice were divided into two groups (*n* = 4). One group of mice was injected with 200 µL D‐Lycosin‐I (9 mg kg^−1^) and the control group was injected intraperitoneally with isopyknic sterile PBS. Note that the day of injection was defined as day 0. The injection was performed every other day for a total of 6 times. In addition, the tumor size and bodyweight of the mice in each group were monitored. The tumor tissues and main organs were fixed in 10% paraformaldehyde and dissected for histological examination. The tumor tissues were stained with the immunofluorescence analysis and the main organs were stained with H&E staining.

### Statistical Analysis

Statistical analysis of the data was performed using GraphPad Prism 9. Data were presented as mean ± SEM. P values were calculated by unpaired two‐tailed t tests or one‐way ANOVA. Bars with different signs indicate significant differences. *P < 0.05, **P < 0.01, ***P < 0.01, and ****P < 0.01compared to control.

## Conflict of Interest

The authors declare no conflict of interest.

## Author Contributions

P.Z., W.L., and Z.Z. contributed equally to this work. All data and the work reported in the paper have been performed or were generated by the authors unless specified in the text. Z.L., P.Z., X.D., and Y.R. performed conception, design of the study, contribution of resources, and acquired funding; P.Z., W.L., Z.Z., M.L., L.S., Y.W., L.W., C.D., K.W., F.L., Y.N., J.Z., Y.Z., and X.L., performed acquisition of data and/or analysis, curation, and interpretation of data; P.Z. and W.L. and performed drafting of the manuscript. All authors did revisions for important intellectual content and approved the final version for publication.

## Supporting information

Supporting Information

## Data Availability

The data that support the findings of this study are available from the corresponding author upon reasonable request.
