# Supplementary material for: A Lipid‐Sensitive Spider Peptide Toxin Exhibits Selective Anti‐Leukemia Efficacy through Multimodal Mechanisms
Source: Adv Sci (Weinh). 2024 Jul 4;11(32):2404937. doi: 10.1002/advs.202404937 (PMC11348133; doi:10.1002/advs.202404937)
Supplement: Supplementary file 1 — Supporting Information [file ADVS-11-2404937-s001.docx]

Supporting Information

**A Lipid-Sensitive Spider Peptide Toxin Exhibits Selective Anti-leukemia Efficacy through Multimodal Mechanisms**

*PengZhang**^1,2,3#^, Wu Luo^1,6#*^, Zixin Zhang^1,2,3#^, Mingchong Lv^1,2,3^, Longkang Sang^1,2,3^, Yuhan Wen^1,2,3^, Lingxiang Wang^1,2,3^,Changhao Ding^1,2,3^, Kun Wu^1,2,3^, Fengjiao Li^1,2,3^,Yueqi Nie^1,2,3^, Jiaoyue Zhu^1,2,3^, Xiaofeng Liu^5^, Yan Yi^4^*, Xiaofeng Ding^1,2,3^*, Youlin Zeng^7^, Zhonghua Liu^1,2,3^**


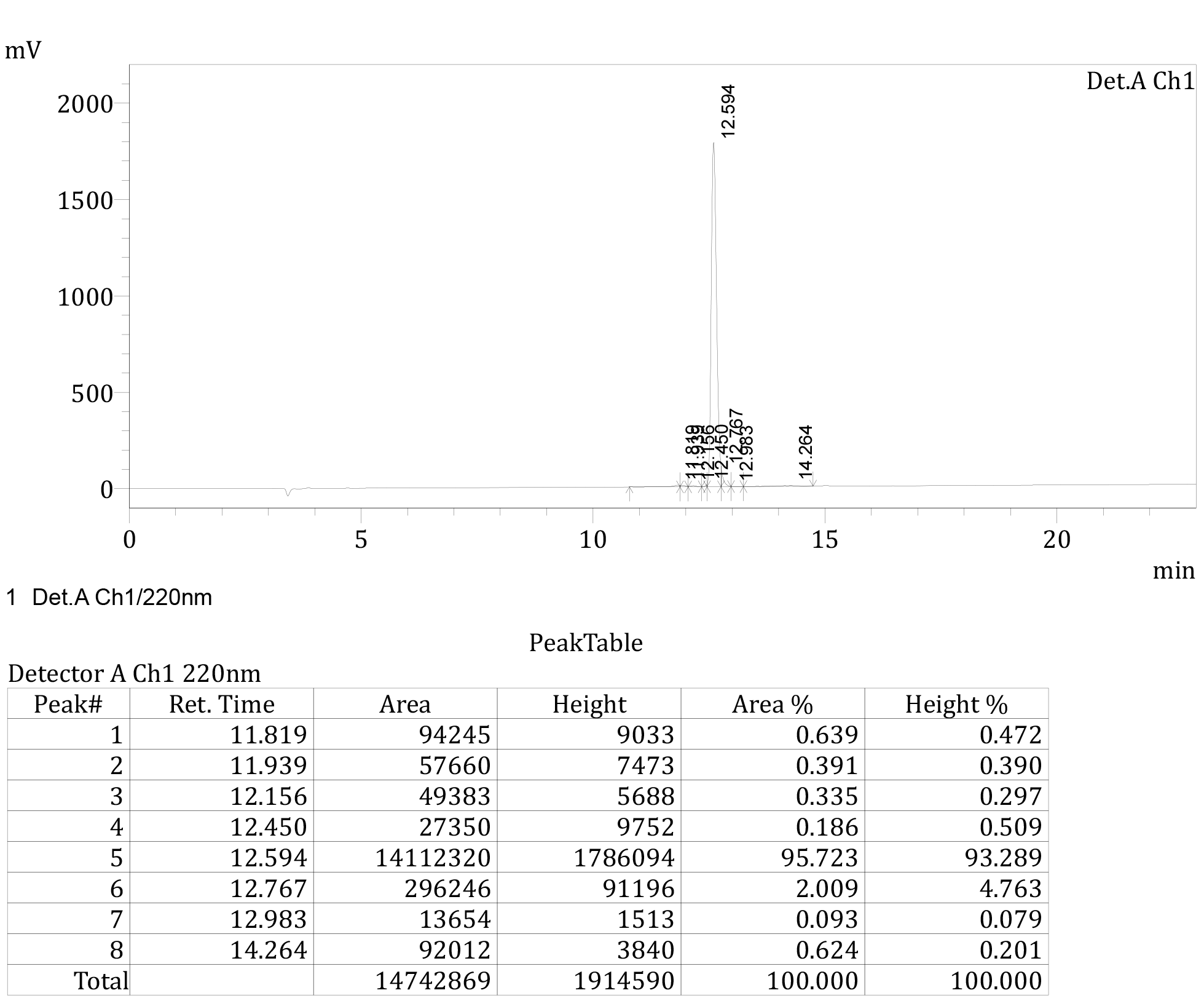


**Figure. S1.** HPLC trace of the peptide Lycosin-I.


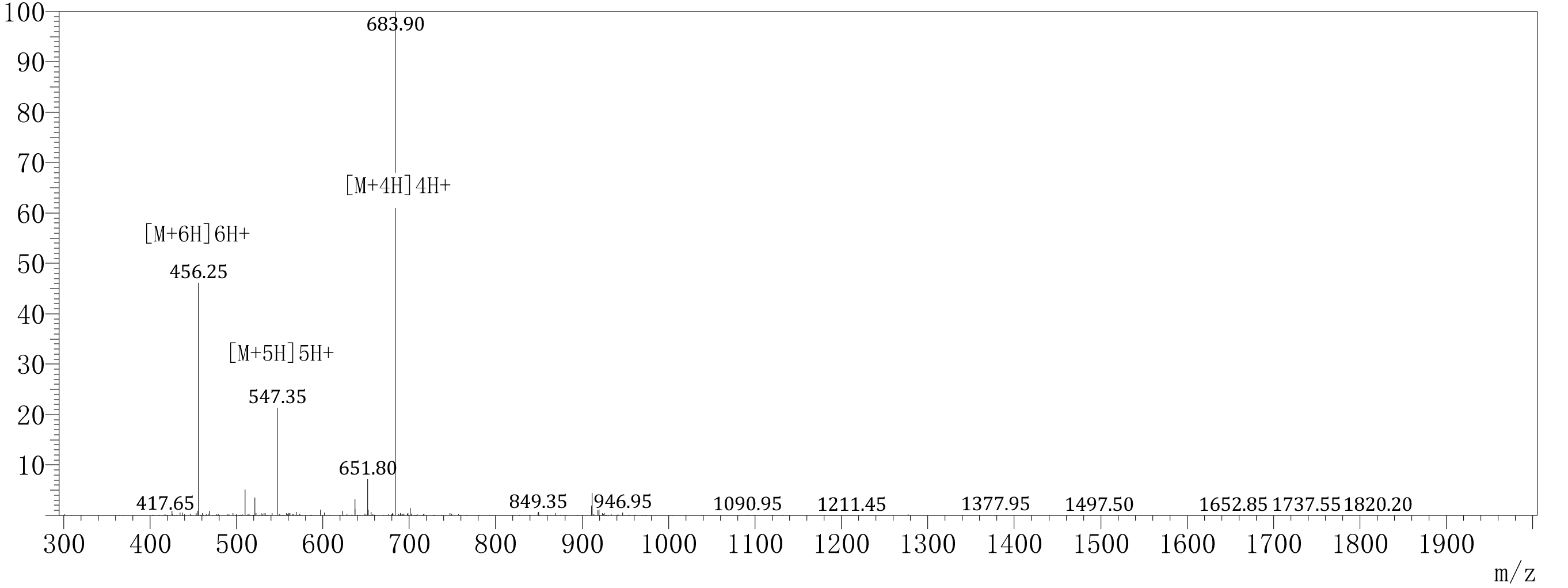


**Figure S2.** Mass spectrum of the peptide Lycosin-I.


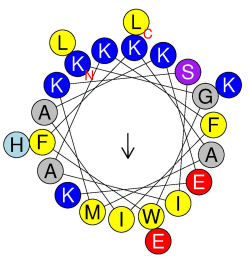


**Figure S3.** The idealized helical wheel projection of Lycosin-I was predicted by Heliquest (https://heliquest.ipmc.cnrs.fr/cgi-bin/ComputParams.py).


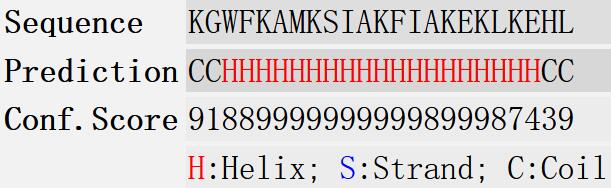


**Figure S4.** Diagram of the secondary structure model of Lycosin-I predicted by i-Tasser software.


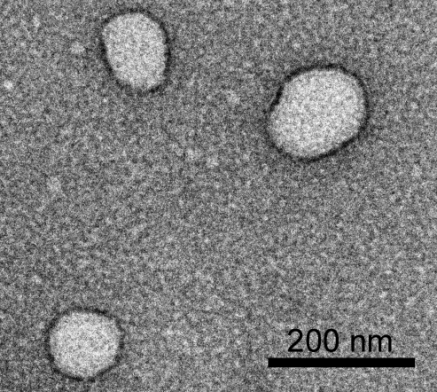


**Figure S5.** TEM images of lipid vesicles formed by POPC/POPS (3:1).


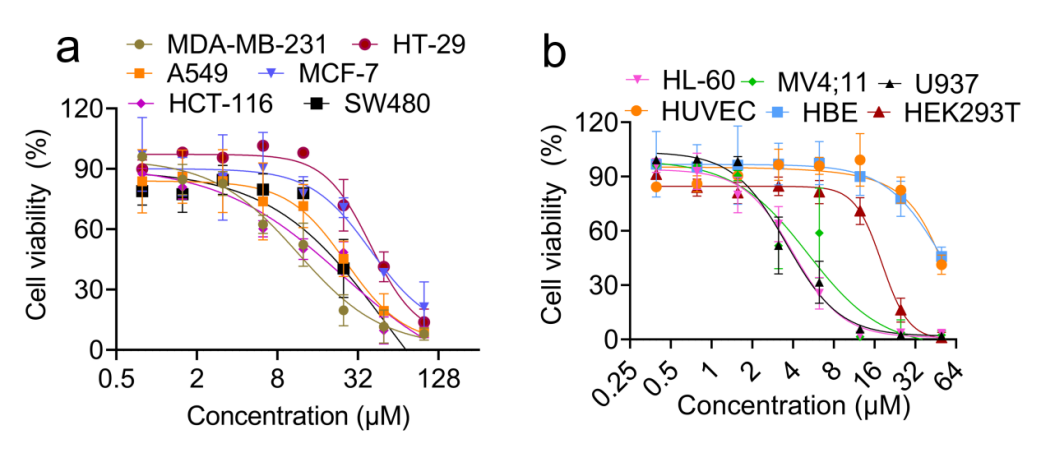


**Figure S6.** The dose-dependent response curves of Lycosin-I on cancer and normal cell lines.


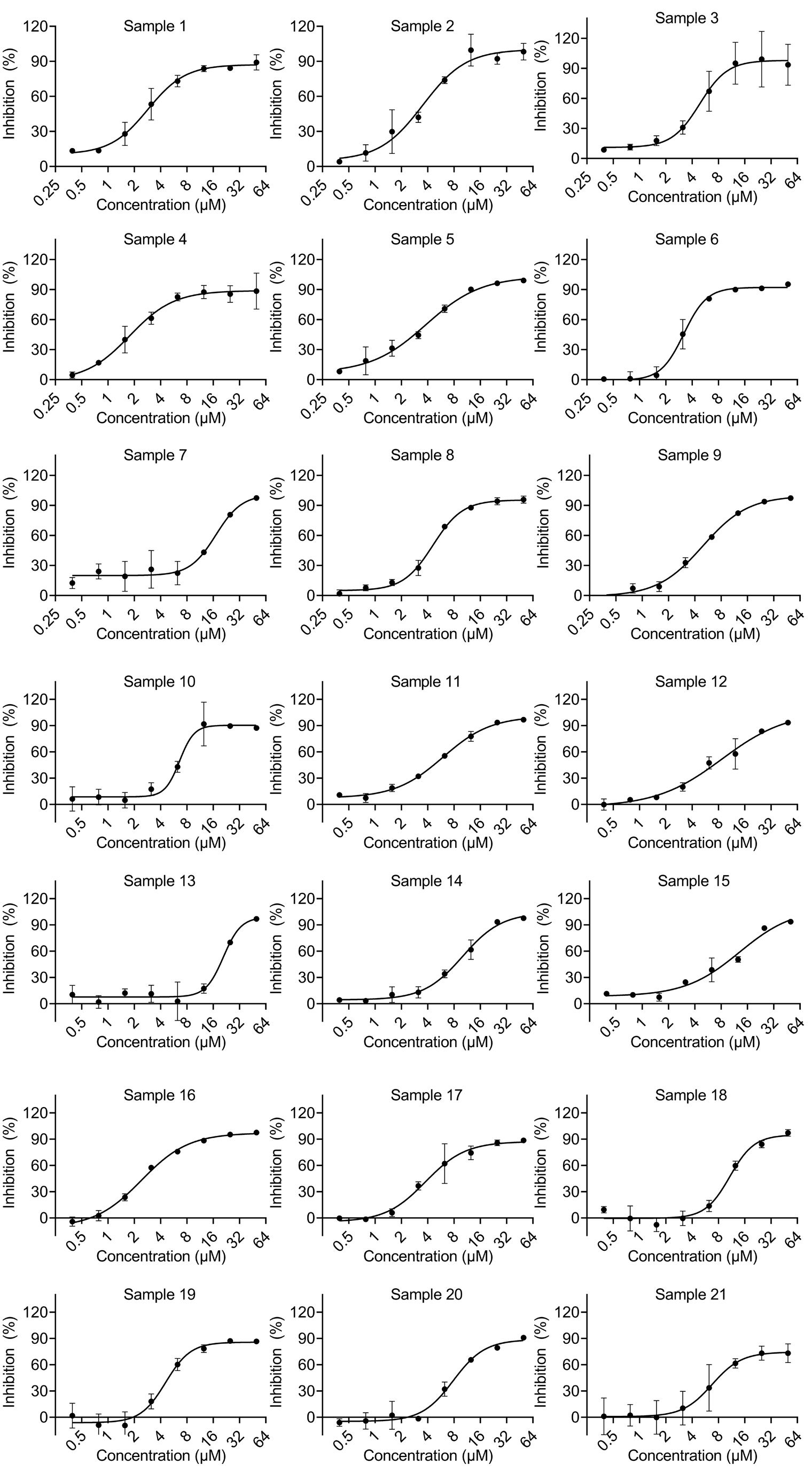


**Figure S7.** The dose-dependent response curves of Lycosin-I on clinical leukemia cells isolated from 21 leukemia patients as described in Table 1.


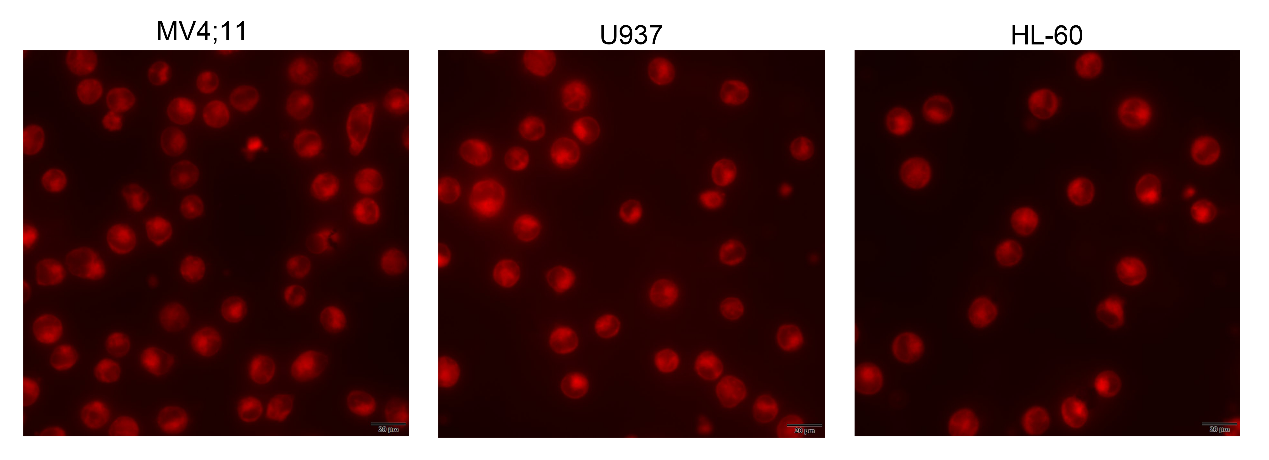


**Figure S8.** The distribution of Cy5-Lycosin-I in MV4;11, U937 and HL-60 cells was detected by fluorescence microscopy. Cells were treated with Cy5-Lycosin-I at 2.5 μM for 1 hour.


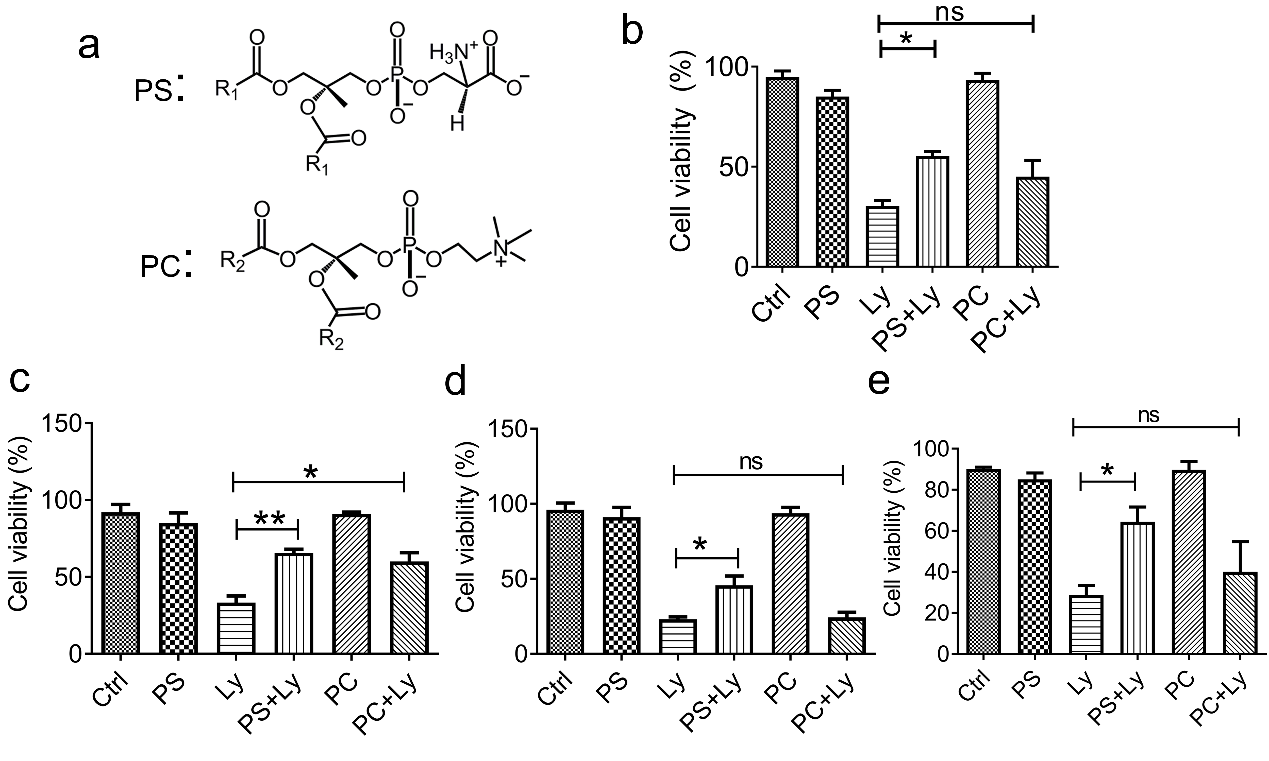


**Figure S9.** Cytotoxic effects of PC and PS liposomes on a variety of cell lines. (a) The chemical structures of PS and PC. R1 and R2 represent the lipid tails of PS and PC, respectively. The cytotoxicity of 10 μM Lycosin-I to K562 (b), MV4;11 (c), U937(d) and HL-60 (e) cells with and without the presence of PS or PC liposomes.


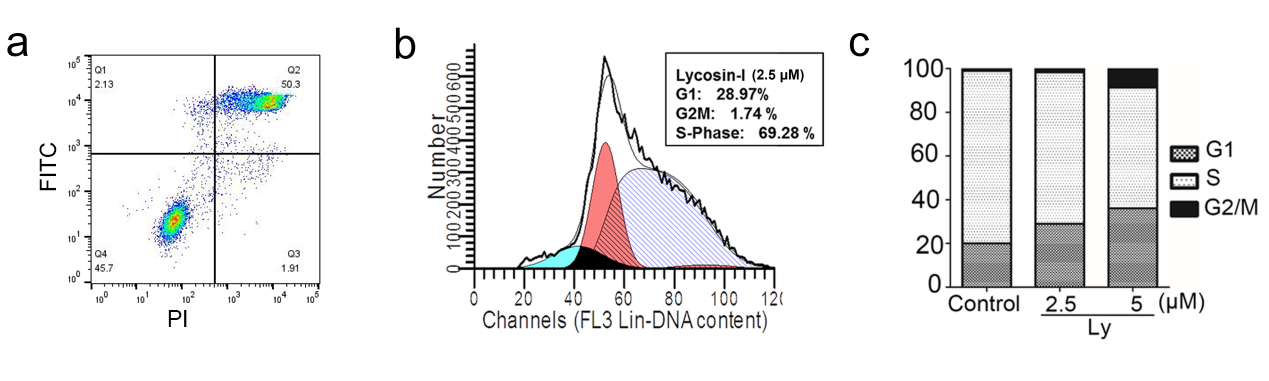


**Figure S10.** Lycosin-I caused apoptosis and cell cycle arrest of K562 cells. (a) K562 cells treated with 2.5 μM Lycosin-I for 24 hours, followed by staining with Annexin-V/PI. The lower right quadrant (Annexin-V/PI) and the upper right quadrant (Annexin-V/PI) indicate the percentage of early apoptosis and late apoptosis. (b) K562 cells were incubated with 2.5 μM Lycosin-I for 24 hours and DNA content was analyzed by flow cytometry. (c) The percentage of K562 cells at G1, S and G2M phases of the cell cycle is shown.


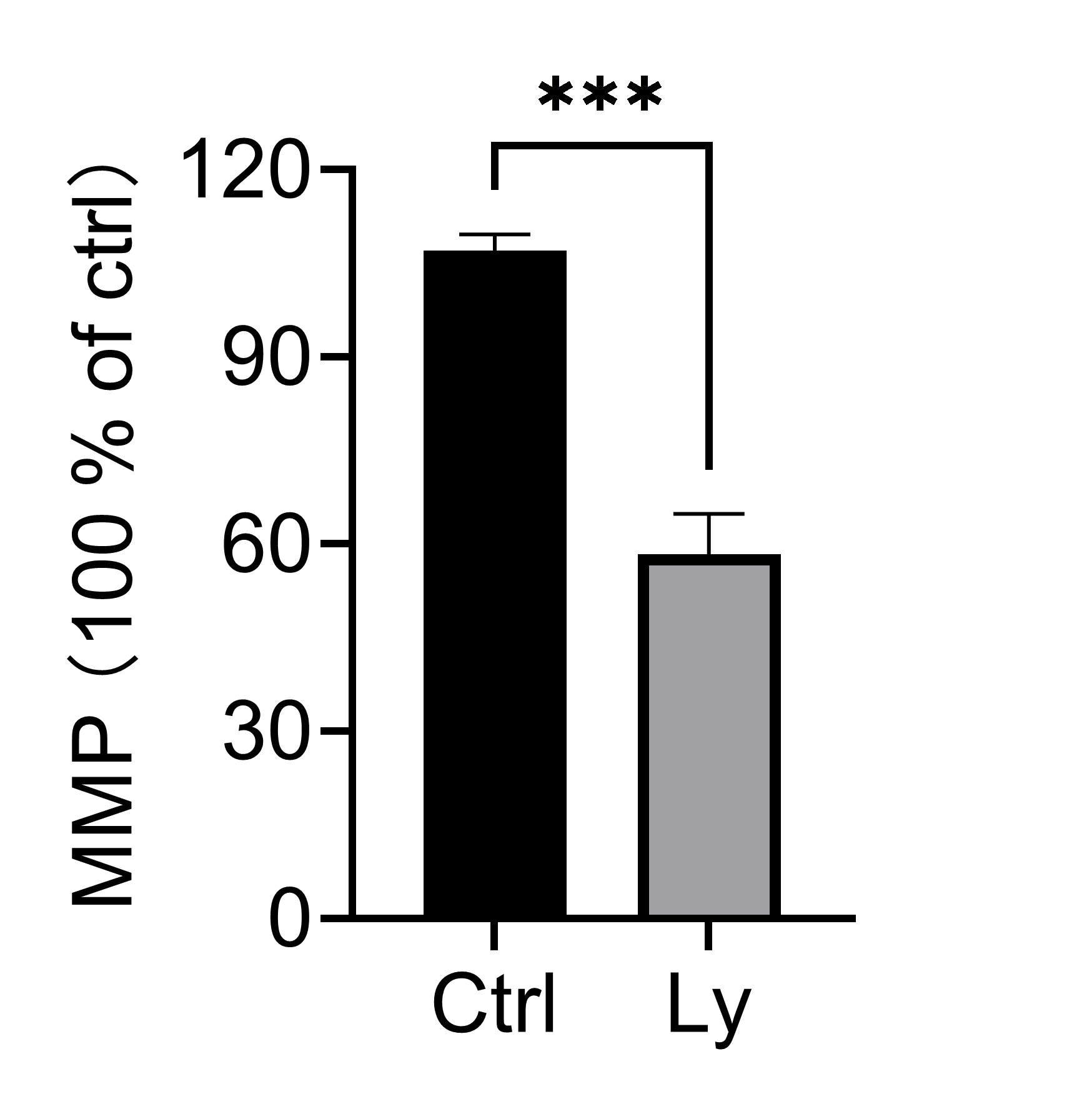


**Figure S11.** Quantitative analysis of MMPs in K562 cells treated with 5 μM Lycosin-I.


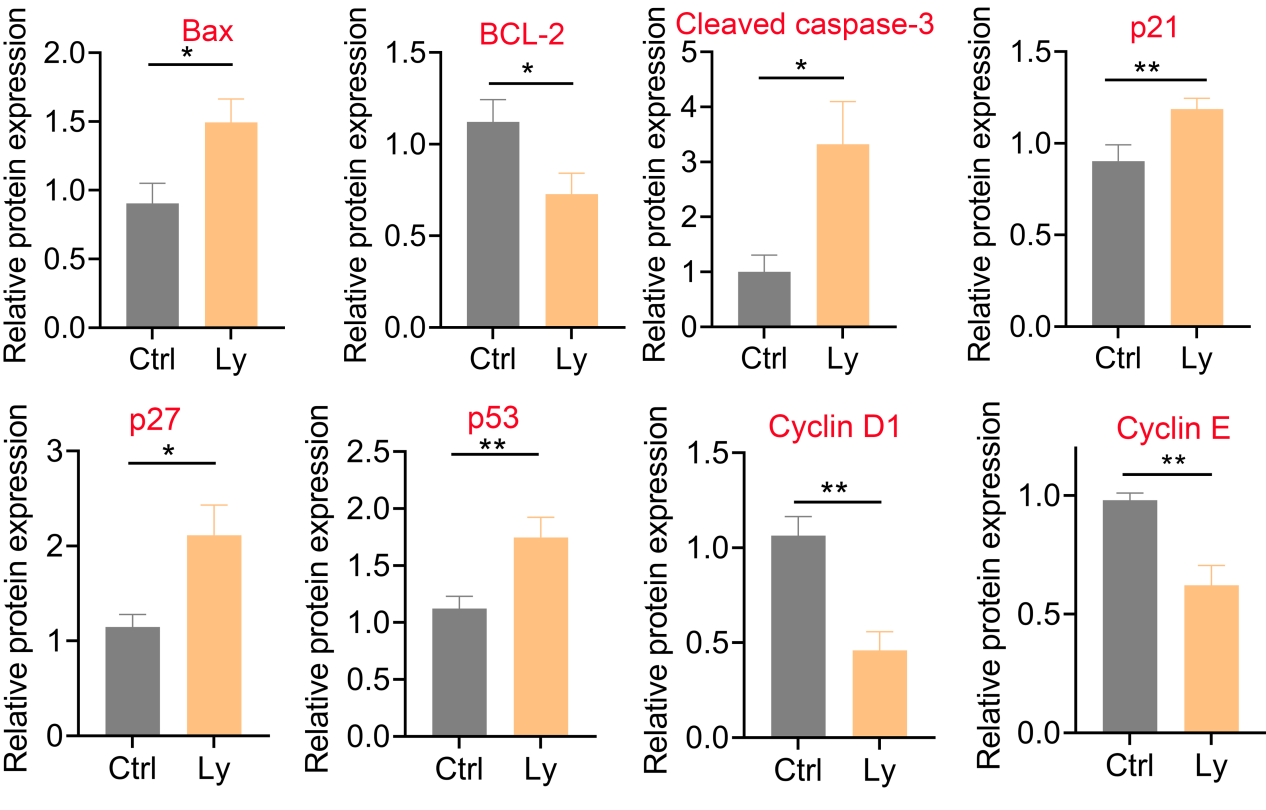


**Figure S12.** Quantitative analysis of protein expression in Figure 3d and 3e by Image J.


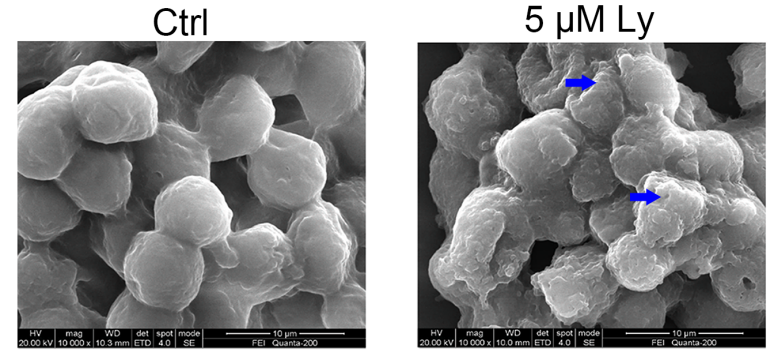


**Figure S13.** Representative SEM images after Lycosin-I treatment. Blue arrows in the figure indicate possible perforations that appear on K562 cells.


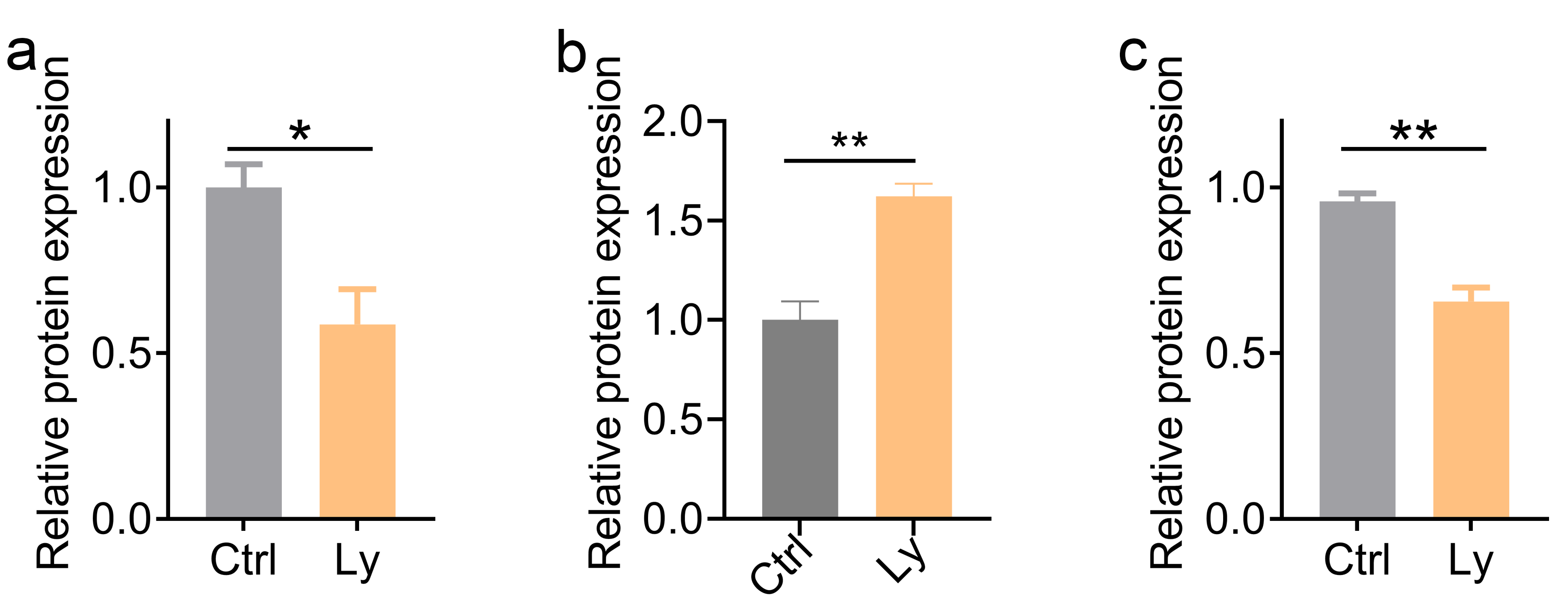


**Figure S14.** Quantitative analysis of protein expression in Figure 4i (a), Figure 4j (b), 4k (c) by Image J.


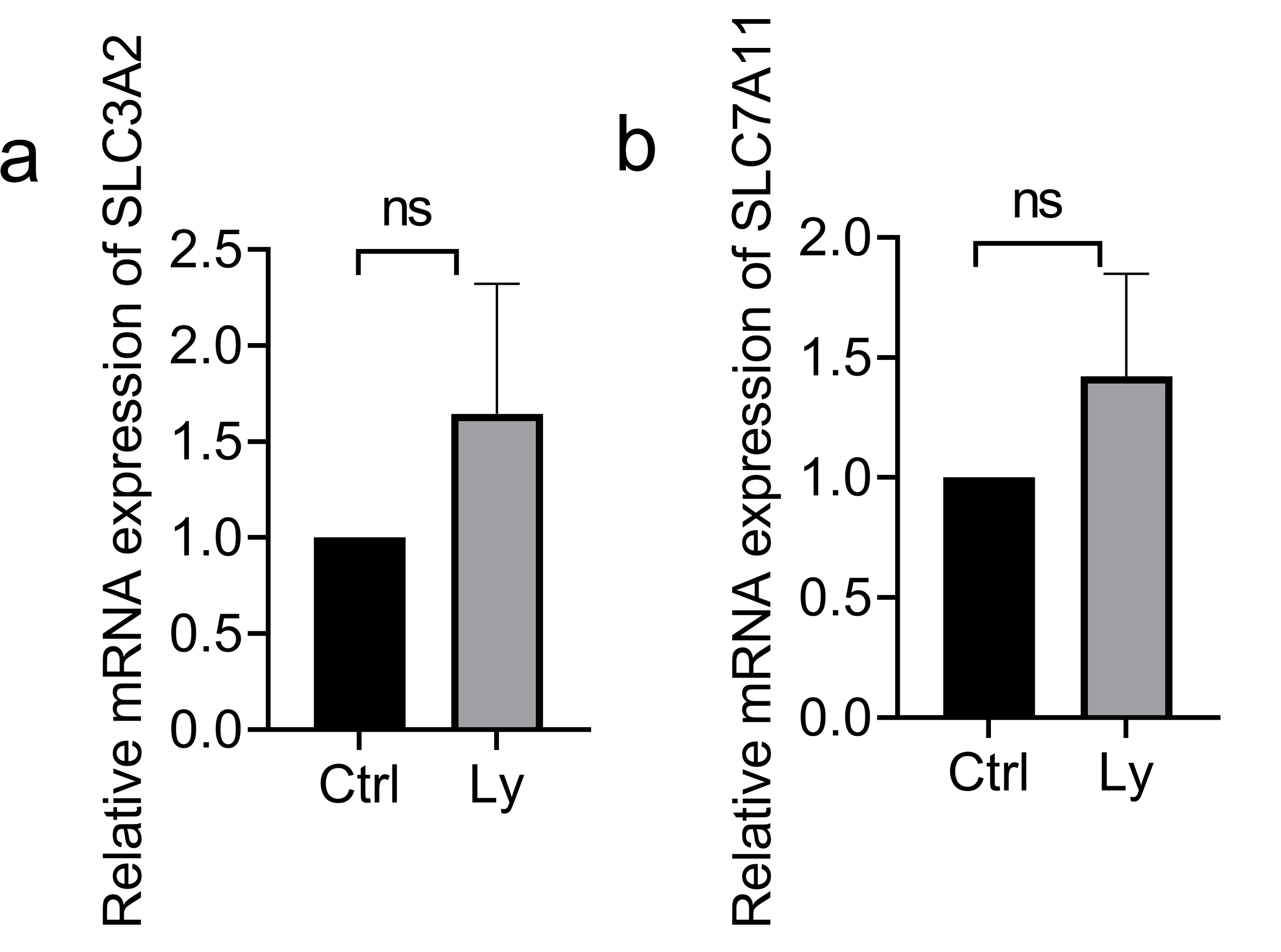


**Figure S15.** The relative RNA level of *SLC3A2* and *SLC7A11* was measured by quantitative real-time polymerase chain reaction after K562 cells were treated with 5 μM Lycosin-I for 24 hours.


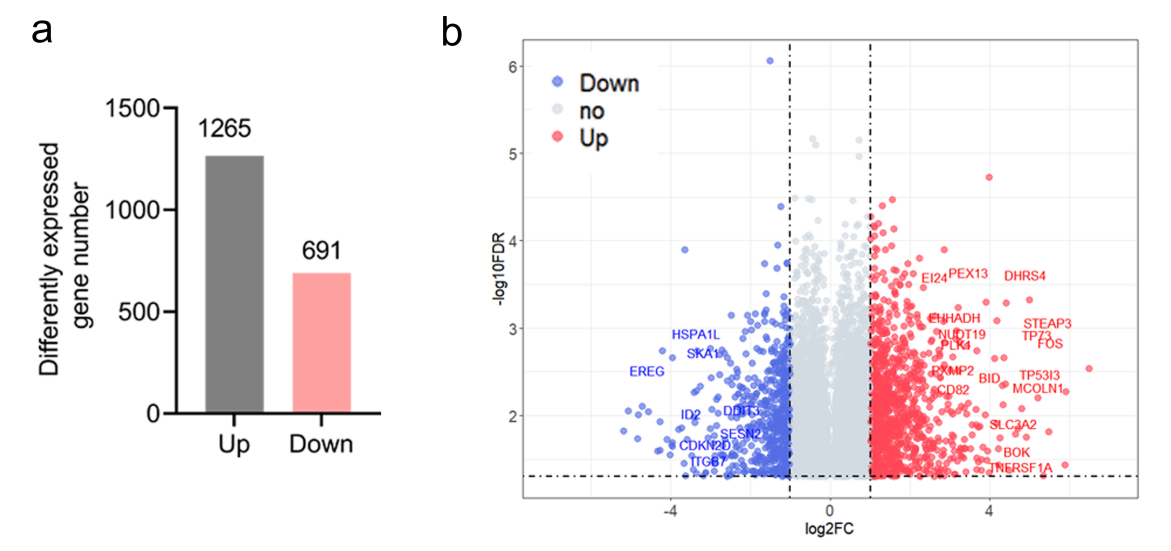


**Figure S16.** Transcriptomics analysis of Lycosin-I-treated K562 cells. (a) DEseq 2 was used to filter the differentially expressed transcripts. There were 1956 differentially expressed transcripts between the 5 μM Lycosin-I-treated and control groups. (b) The volcano plot comparing the mRNA expression profile in the K562 cells: Lycosin-I vs. control. The two horizontal dotted lines correspond to the |Log2FC|>1. The colored dots correspond to the upregulated (red) and downregulated (blue) genes with p < 0.05.


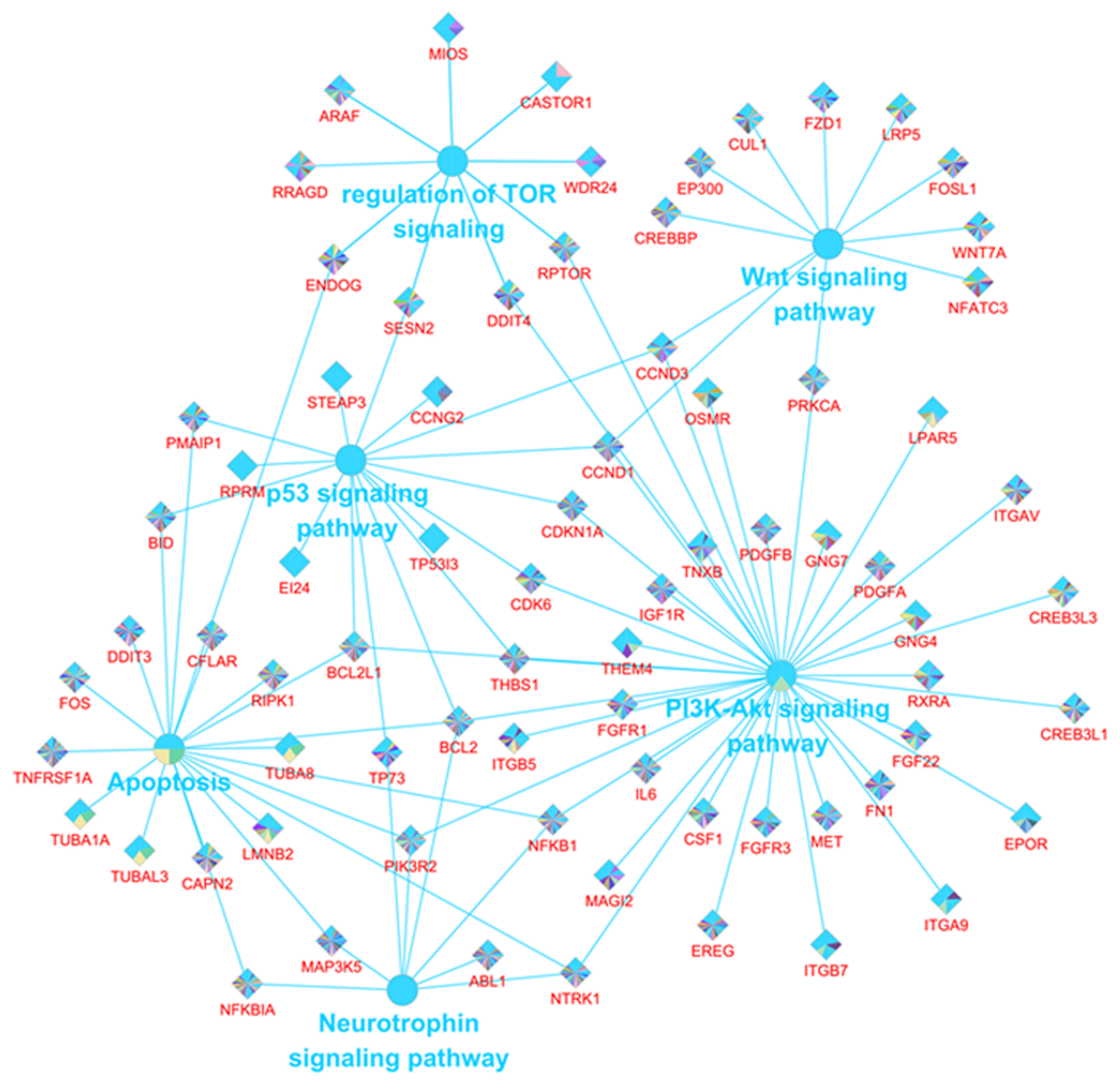


**Figure S17.** PPI and network generated by some of differentially expressed genes in the KEGG signaling pathway.


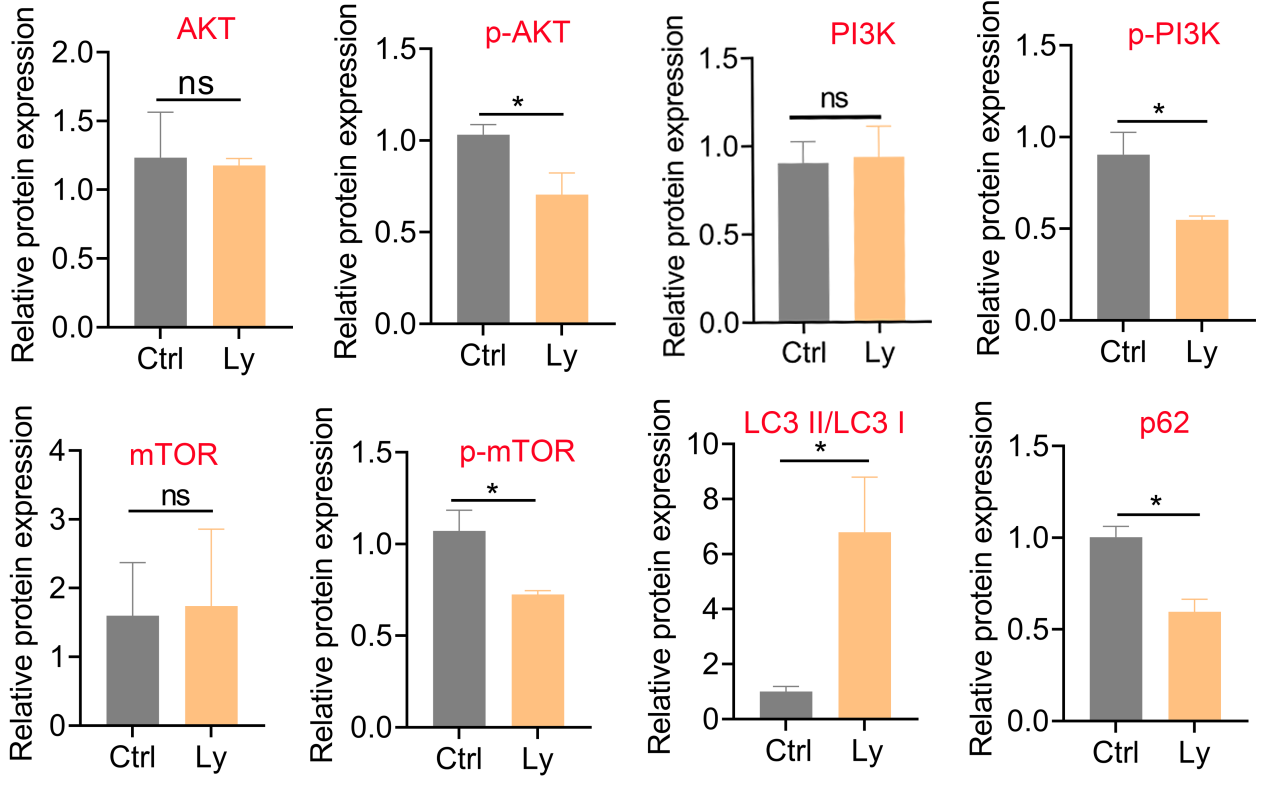


**Figure S18.** Quantitative analysis of protein expression in Figure 5c and 5g by Image J.


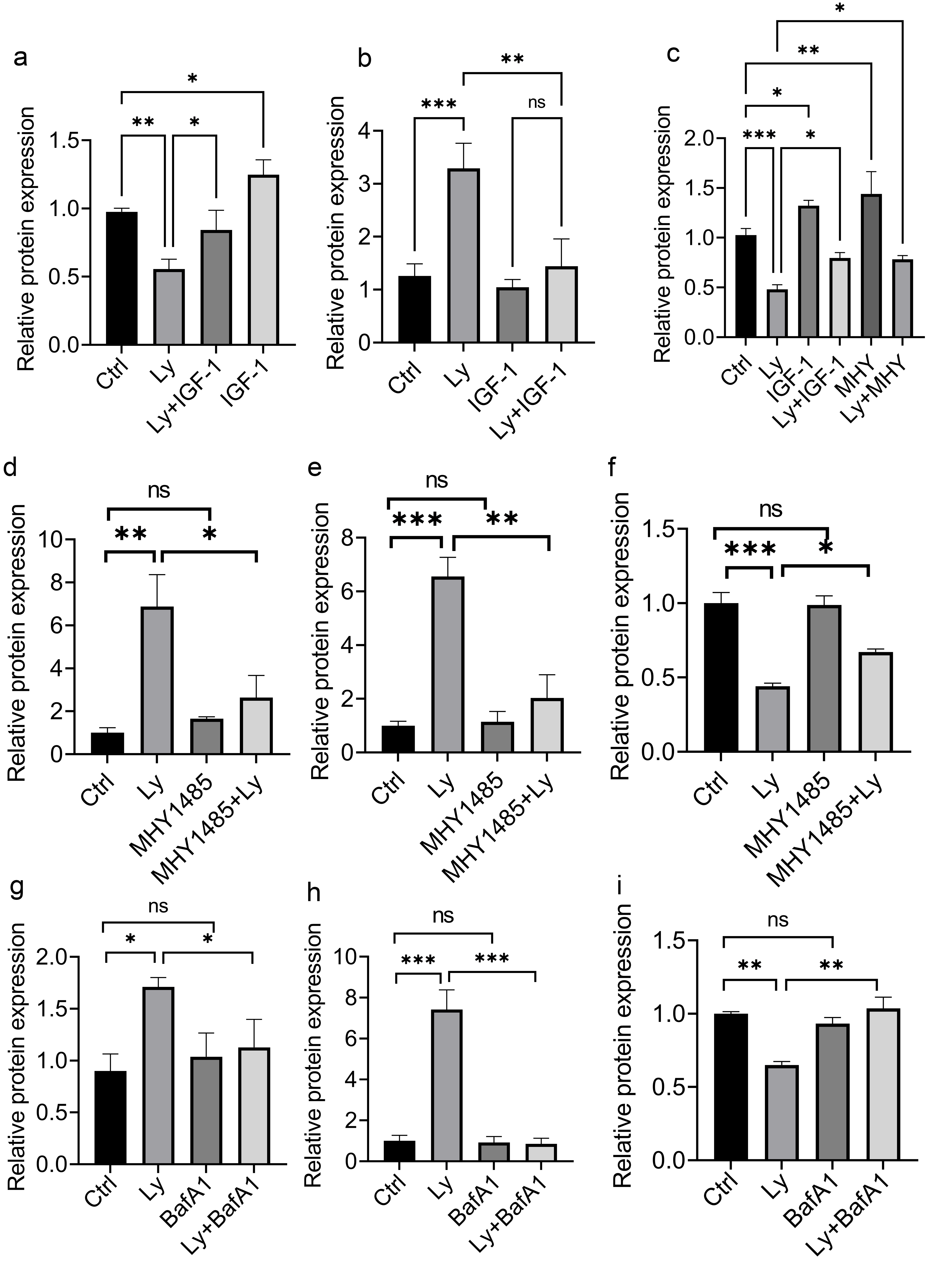


**Figure S19.** Quantitative analysis of protein expression in Figure 5d (a), Figure 5e(b), 5f (c), Figure 5h(d-f) and Figure 5i(g-i) by Image J. d,e and f represent statistical images of protein LC3II/I, cleaved-caspase-3/caspase-3 and GPX4 expression, respectively when cells were treated with MHY1485 plus Lycosin-I. g, h and i represent statistical images of protein LC3II/I, cleaved-caspase-3/caspase-3 and GPX4 expression, respectively when cells were treated with BafA1 plus Lycosin-I.


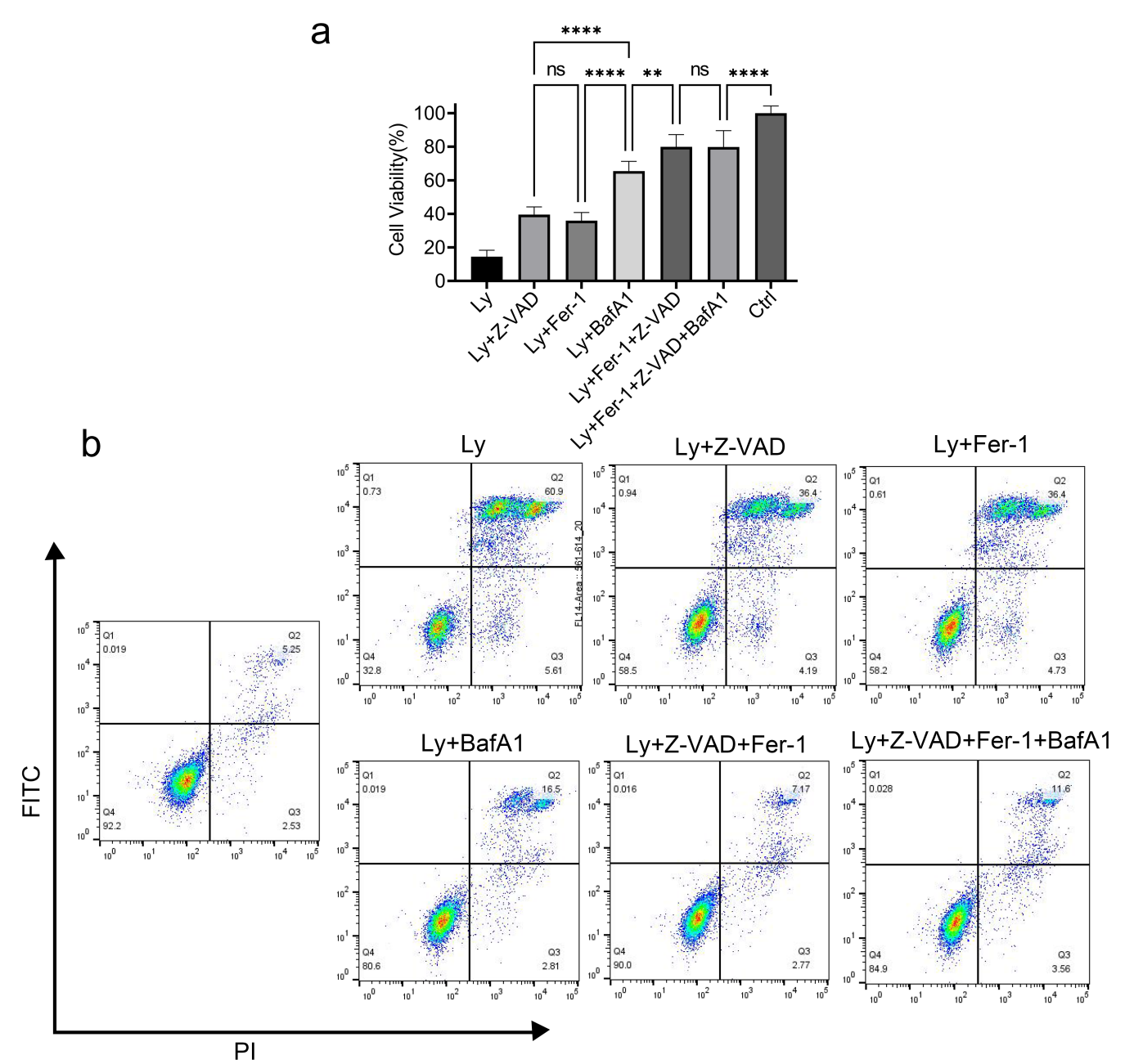


**Fig. S20** (a) Cell viability was tested by CCK-8 assay after the intervention of the apoptosis, ferroptosis, and autophagy by corresponding inhibitors Z-VAD-FMK, Fer-1 and BafA1. Cell viability was tested by flow cytometer after the intervention of the apoptosis, ferroptosis, and autophagy by corresponding inhibitors Z-VAD-FMK, Fer-1 and BafA1. (b) The concentration of Lycosin-I, Z-VAD-FMK, Fer-1 and BafA1was 5μM, 10 μM, 1μM and 40 nM, respectively.

**Figure S21.** HPLC trace of the peptide D-Lycosin-I.

**Figure S22.** Mass spectrum of the peptide D-Lycosin-I.


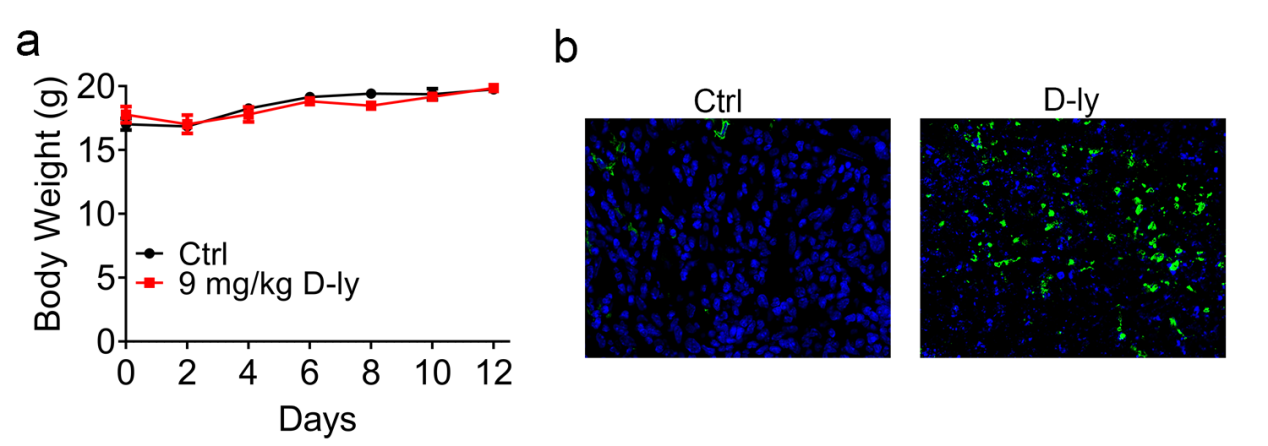


**Figure S23.** (a) Changes in body weight of mice bearing K562 tumors during the study.


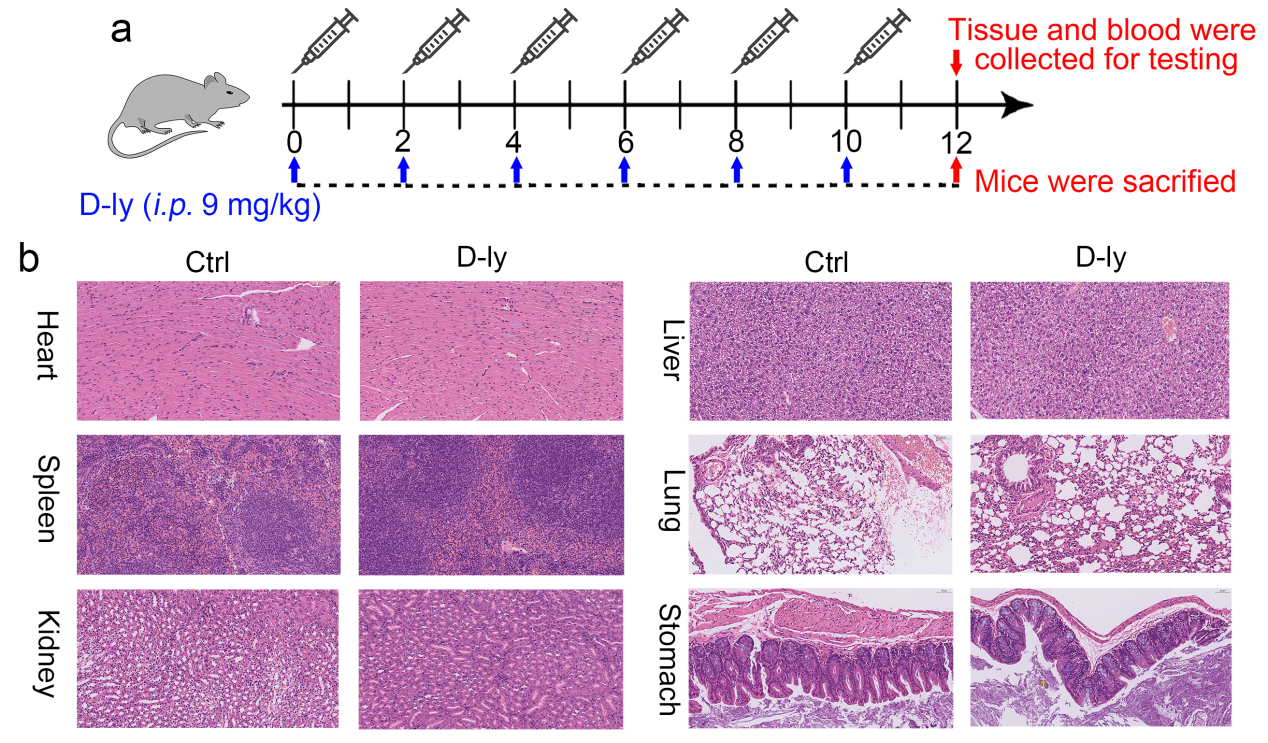


**Figure S24.** *In vivo* safety evaluation of D-lycosin-I. (a) Schematic illustration of the timeline for the *in vivo* safety assessment. (b) H&E staining analysis of mouse heart, liver, spleen, lung and kidney.
